# Supplementary material for: Assessing the potentials of bacterial antagonists for plant growth promotion, nutrient acquisition, and biological control of Southern blight disease in tomato
Source: PLoS One. 2022 Jun 8;17(6):e0267253. doi: 10.1371/journal.pone.0267253 (PMC9176874; doi:10.1371/journal.pone.0267253)
Supplement: S1 File — (DOCX) [file pone.0267253.s001.docx]

**Table S1: Growth of *Stenotrophomonas maltophilia* PPB3 and *Bacillus subtilis* PPB9 on water-yeast broths (WYBs), supplemented with either oxalic acid or citric acid as a carbon source at concentrations of 0.5 mM and 5.0 mM.**

| **Carbon source treatment** | **Bacterial strain** | **Bacterial growth (OD_600_)** | | | | |
| --- | --- | --- | --- | --- | --- | --- |
|  |  | **Day 0** | **Day 1** | **Day 2** | **Day 3** | **Day 4** |
| **Control** | ***Stenotrophomonas* *maltophilia* PPB3** | 0.015±0.003a | 0.019±0.004f | 0.115±0.003e | 0.173±0.003f | 0.174±0.006f |
|  | ***Bacillus* *subtilis* PPB9** | 0.015±0.003a | 0.027±0.004def | 0.118±0.004e | 0.177±0.003f | 0.175±0.003ef |
| **Oxalic acid (0.5 mM)** | ***Stenotrophomonas* *maltophilia* PPB3** | 0.015±0.003a | 0.083±0.009bc | 0.115±0.003e | 0.192±0.004e | 0.199±0.006d |
|  | ***Bacillus* *subtilis* PPB9** | 0.015±0.003a | 0.057±0.003cde | 0.128±0.006e | 0.187±0.003def | 0.199±0.006d |
| **Oxalic acid (5.0 mM)** | ***Stenotrophomonas* *maltophilia* PPB3** | 0.017±0.004a | 0.060±0.006cd | 0.243±0.009b | 0.269±0.007b | 0.309±0.006a |
|  | ***Bacillus* *subtilis* PPB9** | 0.017±0.003a | 0.110±0.006ab | 0.293±0.004a | 0.300±0.006a | 0.323±0.004a |
| **Citric acid (0.5 mM)** | ***Stenotrophomonas* *maltophilia* PPB3** | 0.015±0.003a | 0.083±0.004bc | 0.116±0.003e | 0.192±0.004e | 0.194±0.003de |
|  | ***Bacillus* *subtilis* PPB9** | 0.017±0.003a | 0.063±0.004c | 0.125±0.009e | 0.181±0.006def | 0.187±0.003def |
| **Citric acid (5.0 mM)** | ***Stenotrophomonas* *maltophilia* PPB3** | 0.017±0.003a | 0.107±0.008ab | 0.194±0.009c | 0.247±0.003c | 0.291±0.012b |
|  | ***Bacillus* *subtilis* PPB9** | 0.020±0.000a | 0.131±0.006a | 0.170±0.012d | 0.227±0.003d | 0.267±0.006c |

Values followed by different letters in a column were significantly different (*P<0.005*). Data are mean±SE of three replicates

**Table S2: *In vitro* effect of rhizobacteria on germination and seedling vigour of tomato**.

| **Treatment** | **Germination (%)** | **Total plant length (cm)** | **Vigour Index** |
| --- | --- | --- | --- |
| **Control** | 81.08± 2.40a | 12.34±0.92a | 1000.53±57.50a |
| ***Stenotrophomonas maltophilia* PPB3** | 93.33±2.38b  (15.11%) | 15.30±0.93b  (23.99%) | 1427.95±57.86b  (42.72%) |
| ***Bacillus subtilis* PPB9** | 97.52±1.67b  (20.28%) | 16.31±0.93b  (32.16%) | 1590.45±55.30c  (58.96%) |

Values followed by different letters in a column were significantly different (*P<0.005*). Data are mean±SE of eight replicates. Data in parenthesis indicate increase over control.

**Table S3: *In vivo* effect of rhizobacteria on germination of tomato in pot experiment.**

| **Treatment** | **Germination (%)** | | | | | | | | | | |
| --- | --- | --- | --- | --- | --- | --- | --- | --- | --- | --- | --- |
|  | **Day 2** | **Day 3** | **Day 4** | **Day 5** | **Day 6** | **Day 7** | **Day 8** | **Day 9** | **Day 10** | **Day 11** | **Day 12** |
| **Control** | 0.00±0.00 | 0.00±0.00 | 0.00±0.00c | 5.13±0.94b | 10.41±1.50c | 10.94±2.06c | 18.49±3.26b | 30.31±4.41b | 55.81±5.39b | 60.76±4.61b | 63.25±4.26b |
| ***Stenotrophomonas maltophilia* PPB3** | 0.00±0.00 | 0.00±0.00 | 1.63±0.21b | 5.95±1.20b (15.97) | 15.70±0.75b (50.78) | 18.33±2.13b (67.53) | 55.68±4.79a (201.07) | 72.45±6.31a (139.00) | 78.37±5.07a (36.82) | 79.07±3.36a (29.00) | 76.36±3.43a (25.02) |
| ***Bacillus subtilis* PPB9** | 0.00±0.00 | 0.00±0.00 | 2.45±0.29a | 10.33±1.52a (101.23) | 22.72±3.05a (118.28) | 35.22±3.44a (221.87) | 55.83±5.01a (201.88) | 77.82±5.44a (156.72) | 84.08±5.51a (50.66) | 86.20±4.45a (41.88) | 87.33±4.31a (38.08) |

Values followed by different letters in a column were significantly different (*P<0.005*). Data are mean±SE of three replicates. Data in parenthesis indicate the increase over control.

**Table S4: Percent seedlings with damping-off caused by *Sclerotium rolfsii* in *Stenotrophomonas maltophilia* PPB3 and *Bacillus subtilis* PPB9-treated seedlings of tomato in seed trays**.

| **Treatment** | **Seedlings with damping off (%)** | | | | | | |
| --- | --- | --- | --- | --- | --- | --- | --- |
|  | **Week 1** | **Week 2** | **Week 3** | **Week 4** | **Week 5** | **Week 6** | **Week 7** |
| **Control** | 0.00±0.00 | 6.54±2.14a | 11.56±2.76a | 38.21±3.32a | 45.84±4.01a | 72.92±5.54a | 74.93±6.42a |
| ***Stenotrophomonas maltophilia* PPB3** | 0.00±0.00 | 0.00±0.00b (100.00) | 0.00±0.00b (100.00) | 3.25±1.64bc (91.49) | 5.29±2.22c (88.45) | 15.74±3.56b (78.41) | 17.12±3.43b (77.15) |
| ***Bacillus subtilis* PPB9** | 0.00±0.00 | 0.00±0.00b (100.00) | 0.00±0.00b (100.00) | 8.32±2.68b (78.22) | 19.37±2.51b (57.74) | 23.54±3.65b (67.71) | 26.28±4.11b (64.93) |
| **Provax 200** | 0.00±0.00 | 0.00±0.00b (100.00) | 0.00±0.00b (100.00) | 0.00±0.00c (100.00) | 0.00±0.00c (100.00) | 4.45±1.56c (93.90) | 4.45±1.56c (94.06) |

Values followed by different letters in a column were significantly different (*P<0.005*). Data are mean±SE of three replicates. Data in parenthesis indicate the increase over control.

**Table S5: Development of sclerotia by *Sclerotium rolfsii* in *Stenotrophomonas maltophilia* PPB3 and *Bacillus subtilis* PPB9-treated seedlings of tomato in seed trays**.

| **Treatment** | **Number of sclerotia/cm^2^ surface area** | | | | | | |
| --- | --- | --- | --- | --- | --- | --- | --- |
|  | **Week 1** | **Week 2** | **Week 3** | **Week 4** | **Week 5** | **Week 6** | **Week 7** |
| **Control** | 0.00±0.00 | 1.23±0.62a | 3.55±0.87a | 10.64±1.12a | 11.42±1.11a | 13.33±1.20a | 15.26±1.15a |
| ***Stenotrophomonas maltophilia* PPB3** | 0.00±0.00 | 0.00±0.00b | 0.62±0.21b | 1.14±0.32b | 2.22±0.33b | 2.56±0.32bc | 2.74±0.48bc |
| ***Bacillus subtilis* PPB9** | 0.00±0.00 | 0.00±0.00b | 0.94±0.24b | 1.18±0.36b | 3.64±0.36b | 3.89±0.39b | 4.97±0.63b |
| **Provax 200** | 0.00±0.00 | 0.00±0.00b | 0.00±0.00b | 0.00±0.00b | 0.00±0.00c | 0.50±0.20c | 0.85±0.21c |

Values followed by different letters in a column were significantly different (*P<0.005*). Data are mean±SE of three replicates.

**Table S6: Severity of southern blight disease caused by *Sclerotium rolfsii* in *Stenotrophomonas maltophilia* PPB3 and *Bacillus subtilis* PPB9-treated tomato plants in pots.**

| **Treatment** | **Disease Index (%)** | | | | | | | |
| --- | --- | --- | --- | --- | --- | --- | --- | --- |
|  | **Week 3** | **Week 4** | **Week 5** | **Week 6** | **Week 7** | **Week 8** | **Week 9** | **Week 10** |
| **Control** | 3.54±0.92a | 8.27±2.35a | 39.23±5.01a | 46.84±6.43a | 72.58±6.10a | 75.34±6.31a | 88.84±5.22a | 91.34±4.96a |
| ***Stenotrophomonas maltophilia* PPB3** | 0.00±0.00b  (100.00) | 0.00±0.00b  (100.00) | 4.32±1.04b  (88.99) | 13.34±3.16b  (71.51) | 17.55±4.39b  (75.82) | 19.38±4.80c  (74.28) | 23.28±4.81c  (73.80) | 25.37±4.85c  (72.22) |
| ***Bacillus subtilis* PPB9** | 0.00±0.00b  (100.00) | 0.00±0.00b  (100.00) | 6.25±1.42b  (84.06) | 21.88±4.28b  (53.28) | 27.12±4.30b  (62.63) | 34.10±4.34b  (54.74) | 37.13±4.47b  (58.20) | 40.17±4.70b  (56.03) |
| **Provax 200** | 0.00±0.00b  (100.00) | 0.00±0.00b  (100.00) | 0.00±0.00b  (100.00) | 0.00±0.00c  (100.00) | 0.00±0.00c  (100.00) | 0.00±0.00d  (100.00) | 0.00±0.00d  (100.00) | 0.00±0.00d  (100.00) |

Values followed by different letters in a column were significantly different (*P<0.005*). Data are mean±SE of 15 plants. Data in parenthesis indicate the increase over control.

**Table S7: Development of sclerotia by *Sclerotium rolfsii* in *Stenotrophomonas maltophilia* PPB3 and *Bacillus subtilis* PPB9-treated seedlings of tomato in pots**.

| **Treatment** | **Number of sclerotia/cm^2^ surface area** | | | | | | | |
| --- | --- | --- | --- | --- | --- | --- | --- | --- |
|  | **Week 3** | **Week 4** | **Week 5** | **Week 6** | **Week 7** | **Week 8** | **Week 9** | **Week 10** |
| **Control** | 1.07±0.30a | 1.53±0.51a | 6.07±1.21a | 7.15±1.18a | 11.19±1.54a | 16.33±1.53a | 17.84±1.79a | 21.39±1.70a |
| ***Stenotrophomonas maltophilia* PPB3** | 0.00±0.00b | 0.00±0.00b | 0.60±0.21b | 1.14±0.40bc | 2.36±0.57bc | 3.49±0.78b | 3.69±0.79c | 2.12±0.49c |
| ***Bacillus subtilis* PPB9** | 0.00±0.00b | 0.00±0.00b | 1.44±0.36b | 2.21±0.47b | 4.71±0.56b | 5.88±0.79b | 5.99±0.81b | 5.60±0.77b |
| **Provax 200** | 0.00±0.00b | 0.00±0.00b | 0.00±0.00b | 0.00±0.00c | 0.00±0.00c | 0.00±0.00c | 0.00±0.00d | 0.00±0.00c |

Values followed by different letters in a column were significantly different (*P<0.005*). Data are mean±SE of 15 pots.

**Table S8: Population of *Stenotrophomonas maltophilia* PPB3 and *Bacillus subtilis* PPB9 in the upper (toward stem) (top), middle, and lower (toward root tip) (bottom) segments of 1-, 2-, 3-, 4-, 5-, 6- and 7-week-old tomato seedlings**.

| **Bacteria** | **Root segment** | **Bacterial population (CFU/g root tissues) (×10^7^)** | | | | | | |
| --- | --- | --- | --- | --- | --- | --- | --- | --- |
|  |  | **Week 1** | **Week 2** | **Week 3** | **Week 4** | **Week 5** | **Week 6** | **Week 7** |
| ***Stenotrophomonas maltophilia* PPB3** | **Bottom** | 12.56±1.28bc | 26.73±2.92ab | 31.46±3.23b | 51.36±3.82b | 64.77±3.26b | 71.52±4.75b | 91.35±6.65b |
|  | **Middle** | 7.28±0.72d | 16.91±1.44cd | 23.78±1.70c | 32.44±3.34c | 36.35±2.83d | 43.70±2.92c | 44.43±3.54d |
|  | **Upper** | 5.57±0.91d | 12.84±1.21d | 17.17±1.50d | 28.73±3.32c | 23.51±2.90e | 23.19±3.21c | 29.32±3.48d |
|  | **Mean total** | 25.42 | 56.48 | 72.42 | 112.53 | 124.63 | 138.41 | 165.10 |
| ***Bacillus subtilis* PPB9** | **Bottom** | 23.30±2.48a | 31.78±3.59a | 62.42±4.77a | 69.26±4.51a | 82.54±5.04a | 97.75±7.07a | 139.00±6.93a |
|  | **Middle** | 14.58±1.47b | 21.27±1.89bc | 23.41±1.49c | 32.27±3.44c | 47.34±2.91c | 59.69±3.18b | 65.34±4.54c |
|  | **Upper** | 9.75±1.21cd | 14.39±1.22cd | 21.38±2.70cd | 26.52±3.60c | 31.50±1.61de | 32.89±2.94cd | 35.56±4.64d |
|  | **Mean total** | 47.63 | 67.44 | 107.21 | 128.05 | 161.38 | 190.33 | 239.91 |

Values followed by different letters in a column were significantly different (*P<0.005*). Data are presented as CFU g^−1^ fresh weight, each from three sets of five roots harvested from three plants at each time point.
