# Supplementary material for: Assessing the potentials of bacterial antagonists for plant growth promotion, nutrient acquisition, and biological control of Southern blight disease in tomato
Source: PLoS One. 2022 Jun 8;17(6):e0267253. doi: 10.1371/journal.pone.0267253 (PMC9176874; doi:10.1371/journal.pone.0267253)

PLOS ONE

Assessing the potentials of bacterial antagonists for plant growth promotion, nutrient acquisition, and biological control of Southern blight disease in tomato

--Manuscript Draft--

**Manuscript Number:**	PONE-D-21-13656
**Article Type:**	Research Article
**Full Title:**	Assessing the potentials of bacterial antagonists for plant growth promotion, nutrient acquisition, and biological control of Southern blight disease in tomato
**Short Title:**	Bacterial antagonists for biological control of Southern blight disease in tomato
**Corresponding Author:**	MD. M. Hossain, Ph.D.<br>Bangabandhu Sheikh Mujibur Rahman Agricultural University<br>Gazipur, BANGLADESH
**Keywords:**	Inoculation; rhizobacteria; Solanum lycopersicum; biocontrol; Sclerotium rolfsii
**Abstract:**	Southern blight of tomato caused by Sclerotium rolfsii can cause severe plant mortality and yield losses. The use of rhizobacteria for the biological control of Southern blight disease is a potent alternative to fungicidal management. Although rhizobacteria are prolific candidates, comprehensive reports regarding their use in tomato disease management are limited. The present study screened rhizobacterial strains for antagonism against S. rolfsii in a dual culture assay. Cell-free filtrates of the potential strains were further screened for extracellular antifungal activity and characterized for plant-growth-promoting and biocontrol traits. The promising candidates were tested under in vitro–, greenhouse, and field conditions. Of the strains, Stenotophomonas maltophilia PPB3 and Bacillus subtilis PPB9 displayed the highest antagonism against S. rolfsii in dual culture assay (PPB3 88% and PPB9 71%inhibition), and culture filtrate assay (PPB3 53-100% and PPB9 54-100% inhibition at various concentrations). Oxalic acid production by S. rolfsii was also inhibited by both. The strains produced hydrogen cyanide, chitinases, siderophores, biofilm, and indole acetic acid. They showed the potential to solubilize phosphate and fix nitrogen. Seed treatment with S. maltophilia PPB3 and B. subtilis PPB9 improved seed germination and tomato seedling vigor in vitro. Significant increase in plant growth, chlorophyll contents, and N, P, and K concentrations were attained in bacterized plants compared to controls. Application of antagonists in seedling, potted and field-grown tomatoes significantly controlled symptoms of damping-off and Southern blight. The sclerotial counts decreased significantly in these soils. Bacteria-protected plants were more vigorous and had a higher yield than those in the other treatment categories. Bacteria efficiently colonized the entire roots, and their populations increased significantly in the protected plants. The results show the potential capabilities of S. maltophilia PPB3 and B. subtilis PPB9 for growth promotion, nutrient acquisition, and biocontrol of southern blight disease in tomato.
**Order of Authors:**	Farjana Sultana
	MD. M. Hossain, Ph.D.
**Additional Information:**	
**Question**	**Response**
**Financial Disclosure**<br><br>Enter a financial disclosure statement that describes the sources of funding for the work included in this submission. Review the submission guidelines for detailed requirements. View published research articles from *PLOS ONE* for specific examples.	Ministry of Science and Technology, Bangladesh

Powered by Editorial Manager® and ProduXion Manager® from Aries Systems Corporation

This statement is required for submission and **will appear in the published article** if the submission is accepted. Please make sure it is accurate.<br><br>**Unfunded studies**<br>Enter: *The author(s) received no specific funding for this work.*<br><br>**Funded studies**<br>Enter a statement with the following details:<br>• Initials of the authors who received each award<br>• Grant numbers awarded to each author<br>• The full name of each funder<br>• URL of each funder website<br>• Did the sponsors or funders play any role in the study design, data collection and analysis, decision to publish, or preparation of the manuscript?<br>• **NO** - Include this sentence at the end of your statement: *The funders had no role in study design, data collection and analysis, decision to publish, or preparation of the manuscript.*<br>• **YES** - Specify the role(s) played.<br><br>* typeset	
**Competing Interests**<br><br>Use the instructions below to enter a competing interest statement for this submission. On behalf of all authors, disclose any competing interests that could be perceived to bias this work—acknowledging all financial support and any other relevant financial or non-financial competing interests.<br><br>This statement is required for submission and **will appear in the published article** if the submission is accepted. Please make sure it is accurate and that any funding sources listed in your Funding Information later in the submission form are also declared in your Financial Disclosure statement.<br><br>View published research articles from	The authors have declared that no competing interests exist.

PLOS ONE for specific examples.<br><br>**NO authors have competing interests**<br><br>Enter: *The authors have declared that no competing interests exist.*<br><br>**Authors with competing interests**<br><br>Enter competing interest details beginning with this statement:<br><br>*I have read the journal's policy and the authors of this manuscript have the following competing interests: [insert competing interests here]*<br><br>* typeset	
**Ethics Statement**<br><br>Enter an ethics statement for this submission. This statement is required if the study involved:<br><br>• Human participants<br>• Human specimens or tissue<br>• Vertebrate animals or cephalopods<br>• Vertebrate embryos or tissues<br>• Field research<br><br>Write "N/A" if the submission does not require an ethics statement.<br><br>General guidance is provided below. Consult the submission guidelines for detailed instructions. **Make sure that all information entered here is included in the Methods section of the manuscript.**	N/A

**Format for specific study types**<br><br>**Human Subject Research (involving human participants and/or tissue)**<br>• Give the name of the institutional review board or ethics committee that approved the study<br>• Include the approval number and/or a statement indicating approval of this research<br>• Indicate the form of consent obtained (written/oral) or the reason that consent was not obtained (e.g. the data were analyzed anonymously)<br><br>**Animal Research (involving vertebrate animals, embryos or tissues)**<br>• Provide the name of the Institutional Animal Care and Use Committee (IACUC) or other relevant ethics board that reviewed the study protocol, and indicate whether they approved this research or granted a formal waiver of ethical approval<br>• Include an approval number if one was obtained<br>• If the study involved *non-human primates*, add *additional details* about animal welfare and steps taken to ameliorate suffering<br>• If anesthesia, euthanasia, or any kind of animal sacrifice is part of the study, include briefly which substances and/or methods were applied<br><br>**Field Research**<br><br>Include the following details if this study involves the collection of plant, animal, or other materials from a natural setting:<br>• Field permit number<br>• Name of the institution or relevant body that granted permission	
**Data Availability**<br><br>Authors are required to make all data underlying the findings described fully available, without restriction, and from the time of publication. PLOS allows rare exceptions to address legal and ethical concerns. See the PLOS Data Policy and FAQ for detailed information.	Yes - all data are fully available without restriction

A Data Availability Statement describing where the data can be found is required at submission. Your answers to this question constitute the Data Availability Statement and **will be published in the article**, if accepted.<br><br>**Important:** Stating 'data available on request from the author' is not sufficient. If your data are only available upon request, select 'No' for the first question and explain your exceptional situation in the text box.<br><br>Do the authors confirm that all data underlying the findings described in their manuscript are fully available without restriction?	
**Describe where the data may be found in full sentences. If you are copying our sample text, replace any instances of XXX with the appropriate details.**<br><br>• If the data are **held or will be held in a public repository**, include URLs, accession numbers or DOIs. If this information will only be available after acceptance, indicate this by ticking the box below. For example: *All XXX files are available from the XXX database (accession number(s) XXX, XXX.)*.<br>• If the data are all contained **within the manuscript and/or Supporting Information files**, enter the following: *All relevant data are within the manuscript and its Supporting Information files.*<br>• If neither of these applies but you are able to provide **details of access elsewhere**, with or without limitations, please do so. For example:<br><br>*Data cannot be shared publicly because of [XXX]. Data are available from the XXX Institutional Data Access / Ethics Committee (contact via XXX) for researchers who meet the criteria for access to confidential data.*<br><br>*The data underlying the results presented in the study are available from (include the name of the third party*	All relevant data are within the manuscript and its Supporting Information files.

and contact information or URL).<br>• This text is appropriate if the data are owned by a third party and authors do not have permission to share the data.<br><br>* typeset	
Additional data availability information:	

Powered by Editorial Manager® and ProduXion Manager® from Aries Systems Corporation

Manuscript Click here to access/download;Manuscript;MS.docx

Assessing the potentials of bacterial antagonists for plant growth promotion, nutrient acquisition, and biological control of Southern blight disease in tomato

Farjana Sultana[1] and Md. Motaher Hossain[2*]

[1]College of Agricultural Sciences, International University of Business Agriculture and Technology, Dhaka 1231, Bangladesh

[2]Department of Plant Pathology, Bangabandhu Sheikh Mujibur Rahman Agricultural University, Gazipur 1706, Bangladesh

***Corresponding author**

Email: hossainmm@bsmrau.edu.bd

Orcid ID: 0000-0001-5667-7510

Abstract

Southern blight of tomato caused by *Sclerotium rolfsii* can cause severe plant mortality and yield losses. The use of rhizobacteria for the biological control of Southern blight disease is a potent alternative to fungicidal management. Although rhizobacteria are prolific candidates, comprehensive reports regarding their use in tomato disease management are limited. The present study screened rhizobacterial strains for antagonism against *S. rolfsii* in a dual culture assay. Cell-free filtrates of the potential strains were further screened for extracellular antifungal activity and characterized for plant-growth-promoting and biocontrol traits. The promising candidates were tested under *in vitro*, greenhouse, and field conditions. Of the strains, *Stenotophomonas maltophilia* PPB3 and *Bacillus subtilis* PPB9 displayed the highest antagonism against *S. rolfsii* in dual culture assay (PPB3 88% and PPB9 71%inhibition), and culture filtrate assay (PPB3 53-100% and PPB9 54-100% inhibition at various concentrations). Oxalic acid production by *S. rolfsii* was also inhibited by both. The strains produced hydrogen cyanide, chitinases, siderophores, biofilm, and indole acetic acid. They showed the potential to solubilize phosphate and fix nitrogen. Seed treatment with *S. maltophilia* PPB3 and *B. subtilis* PPB9 improved seed germination and tomato seedling vigor in vitro. Significant increase in plant growth, chlorophyll contents, and N, P, and K concentrations were attained in bacterized plants compared to controls. Application of antagonists in seedling, potted and field-grown tomatoes significantly controlled symptoms of damping-off and Southern blight. The sclerotial counts decreased significantly in these soils. Bacteria-protected plants were more vigorous and had a higher yield than those in the other treatment categories. Bacteria efficiently colonized the entire roots, and their populations increased significantly in the protected plants. The results show the potential capabilities of *S. maltophilia* PPB3 and *B. subtilis* PPB9 for growth promotion, nutrient acquisition, and biocontrol of southern blight disease in tomato.

Keywords: Inoculation; rhizosphere; *Solanum lycopersicum*; biocontrol; *Sclerotium rolfsii*

1 Introduction

Tomato (*Solanum lycopersicum* L.) is affiliated to the Solanaceae family that includes a large number of economically essential vegetables. It was native to South America, and domestication has occurred in Central America. Europeans distributed the tomato from the Americas to other parts of the world during the 16th century. Presently, this plant is extensively grown worldwide and has become a significant vegetable crop. Based on the worldwide production, tomato currently ranks 7th after maize, rice, wheat, potatoes, soybeans, and cassava [1]. The increasing trend in tomato production and consumption is continuing. This is because tomato fruits can be consumed as raw, cooked, and processed and have good palatability. Tomato fruits have a significant role in human nourishment. It is an abundant source of vitamins, minerals, fibers, and various phytochemicals that provide vital human health and immunity benefits against various disorders. Lycopene, a low-calorie antioxidant element, is the primary carotenoid found in tomatoes [2]. Studies show that lycopene helps reduce prostate cancer risk in the rat [3]. Thus, tomato consumption is considered beneficial for the prevention of the development or progression of cancer. Scientific evidence is also available to correlate tomato intake with low cardiovascular disease risks [4-5].

The global demand for tomatoes is increasing due to rapid population growth. Achieving higher agricultural production in current farming practices is a big challenge. Moreover, tomato plants are vulnerable to an extensive range of abiotic and biotic aggravations. Plant pathogens constitute one of the vital biotic intimidations to tomato cultivation affecting both yield and quality. Worldwide, losses due to plant diseases (excluding viruses) are appraised to be approximately 40% of achievable tomato yield without crop protection [6]. Tomato is extremely susceptible to fungal pathogens that are causing early blight, late blights, Fusarium wilt, and southern blight. Southern blight caused by *Sclerotium rolfsii* is a highly devastating disease that is commonly found in subtropics and tropics. *Sclerotium rolfsii* is a soil-inhabiting omnivorous fungal pathogen infecting a wide range of vegetables, including tomato. The fungus persists in the soil or diseased crop debris as sclerotia for years. The soil-borne inoculum of the fungus upsets seed germination and causes damping off. The fungus also infects the

stem at or near the soil line causing necrotic rot. The rapidly developing lesions girdle the stem, leading to wilting of the plant abruptly and permanently [7]. Whitish mycelium and abundant sclerotia develop on rotting tissues. The pathogen can also cause rot of fruit in contact with the soil [8]. The disease often results in devastating crop losses when soil and weather factors are optimum for the fungus.

The application of synthetic fertilizers and fungicides has been perceived as the core management strategy for maintaining proper growth and health in tomato plants. The current intensive agricultural methods often warrant an excessive application of these chemicals in the crop field and amplify the environmental burden, leading to irredeemable risks for the ecosystems and human health. Non-specific chemicals affect the beneficial microbial species in the soil and cause severe intimidations to soil fertility. Persistent chemicals are particularly hazardous and become rapidly concentrated in the food chain. Ingestion or exposure to these chemicals may result in acute or long-term health problems. Inconsistent and repeated application of the same active compound may also lead to developing a fungicide-resistant pathogen population, making it challenging to manage plant diseases effectively [9]. Hence, current chemical-based agriculture has to be replaced with sustainable practices that carriage fewer public health risks and environmental issues. Most of all, the chemical control of *Sclerotium* diseases is exceptionally challenging due to its soil-borne nature. Counter to these complex and inefficacious chemical-based management strategies is the antipathogenic microorganisms. Besides providing various growth benefits to plants, the microbial antagonists are highly efficient in suppressing soil-borne diseases [10-11].

The rhizosphere is a dynamic habitat for large groups of beneficial microorganisms. It is well known that the rhizosphere microorganisms, directly and indirectly, benefit plant growth and development. The rhizobacteria that actively colonize plant roots and enhance plant growth and yield are referred to as Plant growth-promoting rhizobacteria (PGPR) [12]. PGPR are widely used to improve plant growth and to suppress plant diseases biologically. PGPR application in the tomato field is considered an eco-friendly practice that ensures the higher crop productivity of crops and implements a sustainable approach that manages disease without risk. Numerous strains of PGPR have so far been reported with

potential application in agriculture. Although southern blight is a common disease in tomatoes in Bangladesh, there has been limited PGPR use for plant growth enhancement and disease control. In this view, the present study was laid to evaluate the beneficial effects of rhizobacteria on plant growth promotion, nutrient acquisition, and biological control of Southern blight disease in tomatoes.

2 Materials and Methods

2.1 Bacterial collection source

Bacterial strains previously isolated from the cucumber rhizosphere (*Cucumis sativus* L. cv. Baromashi) [12] were used as bio-inoculants in this study. Nutrient broth (NB) was amended with 15% glycerol to maintain the bacterial stock cultures at -20°C. During bioassays, active cultures were obtained by streaking the stock cultures onto the nutrient agar (NA) plates, followed by incubating at 28°C for 48 hours.

2.2 Phytopathogen cultures

The plant pathogens *Rhizoctonia solani* AR-01, *Phytophthora capsici* PPC-1, *Sclerotinia sclerotiorum* PSB-1, and *Sclerotium rolfsii* SR-1 were used in this study. These fungi were taken from the Department of Plant Pathology Stock Cultures, Bangabandhu Sheikh Mujibur Rahman Agricultural University (BSMRAU), Gazipur, Bangladesh. The fungus cultures were stored in potato dextrose agar (PDA) slant at 4°C until use. In order to use in the bioassays, pieces of stock cultures were placed onto PDA plates and incubated at 25±2°C for five days.

2.3 Screening of bacteria for antagonistic activity against *Sclerotium rolfsii* SR-1

2.3.1 *Dual culture assay*

The antifungal activity of five bacterial isolates, *Pseudomonas stutzeri* PPB1, *Bacillus subtilis* PPB2, *Stenotrophomonas maltophilia* PPB3, *B. amyloliquefaciens* PPB4, *B. subtilis* PPB5 and *B. subtilis* PPB9 against *S. rolfsii* SR-1 was determined by the dual culture technique [17]. ~~Overnight cultures of bacteria were streak inoculated~~ on one end of plates containing glucose casamino yeast extract medium (GCY) (1.5% glucose, 0.15% casamino acid, 0.1% yeast extract, 0.15% K_2HPO_4, 0.1% $MgSO_4.7H_2O$, 2.0% agar, pH 7.2). GCY plates amended with

Provax-200 (Carboxin, 5,6-dihydro-2-methyl-1,4-oxathin-3-carboxanilide) (Hossain Enterprise CC Limited, Dhaka, Bangladesh) at a concentration of 200 ppm were included as a positive check. Provax-200 was added in an autoclaved GCY medium following the poisoned-food technique before the fungal inoculation. The GCY plates without bacterial inoculation or fungicidal amendment were treated as negative controls. Later, a mycelial disk (5-mm in diameter) excised from the new and active colony of *S. rolfsii* SR-1 was placed onto the center of the Petri plates. All treatments were prepared in triplicates. The inoculated plates were incubated at 28°C in an incubator. After five days, when *S. rolfsii* SR-1 in control plates covered the entire plate, the fungal radial growth diameter in dual cultures was recorded. The percent inhibition (PI) of *S. rolfsii* SR-1 due to the bacterial antagonistic activity ~~over the control~~ was calculated using the formula of Sundar et al. [21].

$$\% \text{ Inhibition of growth} = \frac{X-Y}{X} \times 100$$

Where,

X = Mycelial growth of the pathogen in the absence of bacteria.

Y = Mycelial growth of the pathogen in the presence of bacteria.

2.3.2 *Culture filtrate assay*

The bacterial isolates listed in this study were further evaluated for antifungal activity of their culture filtrates against *S. rolfsii* isolate SR-1. Bacteria were cultured for five days in Yeast Peptone (YP) broth on a shaker at 120 rpm at 28°C. The broth was centrifuged at 15000 rpm at 4°C, and supernatants were collected. The collected supernatants were passed through 0.45 µm membrane filters. The cell-free filtrates were added to the autoclaved GCY medium at the rate of 0%, 10%, 25%, or 50% (v/v). GCY plates amended with Provax-200 (Carboxin, 5,6-dihydro-2-methyl-1,4-oxathin-3-carboxanilide) (Hossain Enterprise CC Limited, Dhaka, Bangladesh) at a concentration of 200 ppm were included as a positive check. Mycelial plugs of *S. rolfsii* isolate SR-1 from actively growing margin were transferred

onto Petri dishes and incubated at 28°C for five days in an incubator. All treatments were prepared in triplicates. The percentage inhibition of the test pathogen was calculated as described previously.

2.3.3 *Inhibition of oxalic acid production assay*

The efficacy of antagonists was tested against oxalic production by the fungus. An amount of 100 ml potato dextrose broth (PDB) was prepared in 250 ml Erlenmeyer flasks and autoclaved. Each of the flasks was co-inoculated with ten mycelial disks (5-mm in diameter) excised from the fresh colony of *S. rolfsii* SR-1a 9 mm and a loopful of the overnight culture of each antagonist. PDB inoculated with *S. rolfsii* alone served as a control. Three replications were taken for each treatment. The cultures were incubated in an incubator at 28°C for two weeks. The supernatant from each culture was obtained after centrifugation of the broth separately at 4500 rpm for 20 min and filtered by a 0.45 µm Millipore filter. Each cell-free culture filtrate, 5 ml was taken in a 15 ml sterilized centrifuge tube, and 4 ml of calcium chloride acetate buffer was added [13]. The mixtures were centrifuged at 4,500 rpm for 10 min, and the supernatants were thrown away. The deposits were washed with 5 ml of 5% acetic acid saturated with calcium oxalate and recentrifuged. Each deposit was dissolved in 5 ml of 4 N H_2SO_4 in a 100 ml conical flask and heated on a water bath at 80–90°C. Finally, the hot solutions were filtrated with 0.02 N potassium permanganate until faint pink color occured. One ml of 0.02 N $KMNO_4$ reacted with 1.2653 mg of oxalic acid. The oxalic acid production was calculated and expressed as mg/ml [13].

2.4 Characterization of elite bacterial isolates for plant growth promoting and biocontrol traits

2.4.1 Phosphate solubilization

The phosphate solubilization ability of the two bacterial isolates was evaluated using Pikovskaya agar plates [14]. Briefly, bacteria were cultured in NB on a shaker at 120 rpm at 28°C for 48 hours. An aliquot of 10 µL of the bacterial culture was spot inoculated on Pikovskaya agar plates and subsequently incubated in an incubator at 37°C. After six days, the plates were visually checked for a clear zone around the bacterial colonies.

2.4.2 Indole-3-acetic acid (IAA) production

The bacterial isolates were cultured in NB broth amended with 0.1 % tryptophan in an incubator at 37°C for 72 hours. The culture was centrifuged at 15,000×g for 10 min, and the supernatant was

collected. A suspension was prepared by mixing 1 ml of the supernatant with two drops of ortho-phosphoric acid and 4 ml of Salkowski reagent (mixer of 50 ml of 35 % of perchloric acid and 1 ml of 0.5 M $FeCl_3$) and incubated in the dark for 20 min. The absorbance of the solution was measured at 530 nm and plotted on the IAA standard curve.

2.4.3 Nitrogen fixation ability

Bacterial nitrogen fixation ability was assayed following the method described by Ker [15] with slight modifications. Each rhizobacterial strain was cultured in Yeast Extract Peptone broth on a shaker at 120 rpm at 28°C for 24 hours. The culture was diluted to 10^7 CFU mL^{-1}, and an aliquot of 2 μL of the diluted culture was taken for spot inoculation (4 spots $plate^{-1}$) onto N-free solid LG medium [15]. The inoculated plates were incubated at 28°C under static conditions for ten days. The non-inoculated plates incubated at the same condition were used as controls. The growth of colonies in the N-free LG medium designated a positive nitrogen fixation ability of the bacterium.

2.4.4 Siderophore production

Chrome Azurol S (CAS) assay was used to determine the siderophore production ability of the bacteria [14]. In brief, the CAS agar plates were prepared, and each plate was divided into four equal sectors. Each sector was spot inoculated with 10 μL of the bacterial culture (4 spots $plate^{-1}$). The plates were incubated in an incubator at 37°C for 12 days. The formation of orange halo areas around the bacterial colonies on the blue background indicated a positive reaction for siderophore production by the bacteria.

2.4.5 Chitinase Production

Bacteria were cultured in NB overnight on a shaker at 120 rpm at 28°C. Chitin plates were prepared by amending M9 agar medium with 1 % (w/v) colloidal chitin. The plates were divided into four equal sectors, and each sector was spot (4 spots $plate^{-1}$) inoculated with 10 μL of overnight grown culture. The inoculated plates were incubated at 37°C for 96 hours. The formation of the clear zone around bacterial colonies implied a positive reaction for chitinase production [16].

2.4.6 Hydrogen cyanide (HCN) production

Bacteria were cultured in NB overnight on a shaker at 120 rpm at 28°C. Nutrient agar was prepared by supplementing with 0.44% (w/v) of glycine and plated in 9 cm Petri dishes. The agar surface was streak-inoculated with the bacterial culture. A sterile Whatman filter paper No. 1 drenched in filter sterile 2%

(w/v) sodium carbonate in 0.5% (v/v) picric acid was placed on top of the culture. The plates were incubated in an incubator at 30°C for 72 hours. The change in color of the overlaid filter paper from yellow to orange, red, or brown implied lesser, moderate, or higher HCN production levels, respectively.

2.4.7 ACC Deaminase Assay

Dworkin and Foster (DF) minimal salts supplemented with 1-aminocyclopropane-1-carboxylic acid (ACC) as the only nitrogen source were prepared [12]. The bacteria were cultured overnight in NB medium and collected by centrifugation. The bacteria were then inoculated into the DF-ACC medium and incubated at 30°C at 160 rpm for 48 hours. The uninoculated DF-ACC medium was used as control. The culture was centrifuged at 15,000×g at 4°C, and then the supernatant was diluted in test tubes with a DF medium at a 100:1 ratio. Two milliliters of ninhydrin reagent were added to each tube. The tubes were shaken and placed in a hot water bath for 30 min. The solution then turned purple. The boiled sample was left at 30°C for another 10 min before measuring the absorbance at 570 nm. In this assay, the DF-ACC medium was used as a blank.

2.4.8 *In vitro* antagonistic activity against wide spectrum of phytopathogens

The dual culture technique was employed to assess the *in vitro* antagonism of the selected antagonists against several phytopathogens, *Rhizoctonia solani* AR-01, *Phytophthora capsici* PPC-1, and *Sclerotinia sclerotiorum* PSB-1 [17]. The mycelial plugs of 5-mm diameter were obtained from the 5-day old culture of these pathogens and individually placed onto the center of the PDA plate. An overnight bacterial culture was then streak inoculated at an equidistance of 3 cm from the fungal plug. The dual inoculated plates were placed in an incubator and incubated at 28°C for seven days. The control cultures of phytopathogens were grown without bacteria. The plates were incubated at 28°C at 28°C for 3 d in an incubator. Inhibition of the fungal mycelial growth in dual culture with bacteria relative to control indicated the antagonistic activity of the bacteria.

2.4.9 *In vitro* biofilm formation

The bacterial ability to produce biofilm was tested during *in vitro* growth of the bacteria. The isolates were grown in NB at 28°C on a shaker at 180 rpm overnight. The cultures were diluted to a ratio of 1:100. Fifty microliters of the diluted culture were inoculated into borosilicate glass test tubes

containing 5 mL of SOBG [18]. The inoculated glass tubes were incubated at 28°C in static condition for 72 hours. The pellicle attached to the broth surface was gently harvested from the glass tubes, washed thrice with sterile distilled water, and quantified at 600 nm [18].

2.5 Inoculum preparation of bacteria for tomato seed treatment

Bacteria were cultured in 250 ml Erlenmeyer flasks containing 100 ml Yeast Peptone (YP) broth on a shaker at 120 rpm overnight at 28°C. The broth was centrifuged at 15000 rpm at 4°C, and bacterial pellets were collected. Each pellet was washed thrice with sterile distilled water. The purified pellet was suspended in 1.5 ml sterile distilled water by vertexing for a few seconds. Approximately 0.65 g tomato seeds (200 seeds) were surface sterilized with 5% (v/v) sodium hypochlorite solution (NaClO) for 3 min, followed by several washes in sterile water and air-dried. Dry seeds were soaked in the bacterial suspension and coated with bacterial cells by frequent stirring for 5 min. Seeds treated with sterile distilled water served as a control. The bacteria-coated seeds were scattered on a Petri dish and air-dried overnight at 25±2°C. The number of bacteria on the seed surfaces was counted by serial dilution technique before sowing.

2.6 Inoculum preparation of bacteria for tomato root treatment

Bacteria were cultured in 250 ml Erlenmeyer flasks containing 100 ml YP broth on a shaker at 120 rpm overnight at 28°C. The broth was centrifuged at 15000 rpm at 4°C, and bacterial pellets were collected. Each pellet was washed twice and resuspended in distilled water. The bacterial suspension was diluted until the final concentration of the bacteria was adjusted to approximately 10^8 CFU mL^{1} (OD_{600} = 0.12).

2.7 *In vitro* effect of bacteria on seed germination and vigor index in tomato

In order to determine the effect of selected bacteria on germination and seedling vigor, 200 bacteria-coated seeds of tomato cv. Mintoo Super F1 Year-round (Lal Teer Seed Company, Gazipur, Bangladesh) were tested. As a control treatment, an equal number of seeds treated with sterile water was prepared. Seeds were placed on two moistened filter paper layers in 9-cm Petri dishes, allotting 25 seeds in each plate. The Petri dishes were incubated at 28±2°C in a light incubator. Sterile water was added to the Petri dishes every other day. The germination percentage and seedling length were recorded

after seven days. The seedling length was measured from the root tip to the shoot tip. Seedling vigor index was calculated from the following formula:

Vigor index = % germination × total plant length

2.8 *In vivo* effect of bacteria on the germination and growth of tomato

The selected rhizobacterial isolates (*S. maltophilia* PPB3 and *B. subtilis* PPB9) were tested for their ability to promote growth in tomato plants. Tomato seeds were treated with bacteria and water (control) as described above. Field soil was used as a potting medium. The texture of the soil was sandy loam, and the pH was 6.38 with 1.08% organic carbon, 1.87% organic matter, 0.27% nitrogen (N), 0.09% phosphorus (P), and 0.87% potassium (K). The soil was autoclaved twice at 24 h intervals at 121°C and 15 psi for 20 minutes. For each treatment, pots (11.50 cm × 15.0 cm) were taken as decuplicate (15 pots). Each pot was filled with about 500 g of the sterilized soils and sown with five seeds. Pots were placed in a growth room at 24°C temperature and a 16/8 h photoperiod for 42 days. The germination percentage in each treatment was recorded daily for 12 days. On day 13, seedlings were thinned to one per pot. Plants were watered on alternate days. Before harvest, three plants were randomly selected for each treatment to determine specific leaf area (SLA) and photosynthetic pigments. Three mature leaves per plant were scanned and weighed to determine the SLA = area (m^2)/dry weight (kg). Leaf area was calculated with the software ImageJ (v1.47). For the determination of leaf chlorophyll content, fresh leaf samples were collected from the same plants and washed with distilled water. Leaves were blotter dried and cut into about ~1 cm piece. One-gram leaf pieces were placed in 5 ml of 80% acetone (v/v) and incubated overnight in the dark. On the next day, the mix was agitated on a shaker until the leaves were completely bleached. The supernatant was collected by centrifuging the solution for 10 minutes at 5000 rpm, which was used to measure total chlorophyll (a+b) at 663 and 645 nm absorbance. Pigment concentrations were calculated using the formula [19] given below:

Total Chlorophyll (a+b) (mg/g FW) = [20.2× (A645) -8.02× (A663)] × V/ (1000×w)

Where,
V = Final volume of 80% acetone (ml)
w = Fresh weight of sample taken (g)

At harvest, the whole plant was gently uprooted, and the roots were washed in running tap water to remove the attached soil. Data were recorded on morphological parameters such as root and shoot

length, the number of leaves, plant diameter, and shoot and root fresh and dry weights. For taking the dry weight, shoots and roots were oven- dried at 65°C for four days and then weighed.

2.9 Determination of nutrient elements

The oven-dried shoots were separated, ground, and prepared in triplicate. After nitric- perchloric acid (7:3) digestion of the shoot samples, the concentration of cations (Na^+, K^+, Ca^{2+}, and Mg^{2+}), iron, and total phosphorus was determined using inductively coupled plasma (ICP) spectrometry (Optima 4300DV, Perkin- Elmer, UK). Nitrogen concentration in the tomato shoots was determined using the Kjeldahl method [20].

2.10 Preparation of wheat grain inoculum of *Sclerotium rolfsii* SR-1

Inoculum of *S. rolfsii* SR-1 was prepared by taking 100 g moistened wheat grain in a 500 ml Erlenmeyer flask. After plugging the mouth with cotton, the conical flasks were autoclaved at 121°C and 15 PSI for 20 min and allowed to cool on a clean bench. The grains were inoculated with 12 to15 mycelial disks (5 mm diameter) obtained from the actively growing margin of 5-day-old PDA cultures of *S. rolfsii* SR-1. The inoculated flasks were plugged with sterile cotton and incubated at 25°C in the dark for 2 to 3 weeks. The flasks were shaken weekly to allow for consistent growth of *S. rolfsii*. The culture continued until the fungus thoroughly colonized the surface of wheat grains. Colonized grains were air-dried at laboratory temperature (25±2^{O}C) and stored at 4°C until further use.

2.11 Biocontrol efficacy of PGPR against *Sclerotium rolfsii*

2.11.1 *Seedling test*

Tomato seeds were surface sterilized by immersing in 5% (v/v) sodium hypochlorite for 3 min, followed by three rinses with sterile distilled water. Bacterized seeds were prepared as described above. The seed treatment with Provax-200 was done at a concentration of 0.3% (w/w). Seeds lacking treatment with the antagonists and fungicide were considered control (unprotected control). Three seed trays (20×10×5 cm) were prepared for each treatment. Autoclaved soil was mixed thoroughly with colonized wheat grain inoculum of *S. rolfsii* at a concentration of 2.0% (w/w), loaded in the trays (1.0 kg/tray), and moistened to approximately 50% water-holding capacity. After three days, each tray was sown with 30 tomato seeds, 10 in each row. Seed trays were placed in a growth room at 24°C temperature and a 16/8 h photoperiod for seven weeks. Plants were watered on alternate days. Seedlings were observed weekly

for damping-off symptoms and signs of *S. rolfsii* until seven weeks after sowing. The incidence of damping-off in seedlings was expressed as a percentage of the total seedlings with damping-off calculating from the following formula:

$$\text{\% Seedlings with damping off} = \frac{(\text{No. of the seedlings with damping off}) \times 100}{(\text{Total no. of seedlings in the tray})}$$

Additionally, the number of sclerotia formed on soil surfaces was counted and expressed per cm^2 surface area.

2.11.2 *Potted plant tests*

In this experiment, treatments included were a control (unprotected), a fungicide protected check, and two PGPR treatments. Surface sterilized tomato seeds were sown in seed trays (20×10×5 cm), and seedlings were raised for two weeks in the net house. Autoclaved field soil was mixed thoroughly with colonized wheat grain inoculum of *S. rolfsii* at a concentration of 2.0% (w/w) and loaded in the pots (11.50 cm × 15.0 cm) (500 g/pot). Fifteen pots were prepared for each treatment. Seedlings were uprooted from seed trays, and bacterial treatment of the seedlings was given by dipping roots in the suspension (10^8 CFU mL^{-1}) of *S. maltophilia* PPB3 and *B. subtilis* PPB9 for 30 min. Seedlings of unprotected control were dipped in the sterilized water. Soils drenched with Provax 200 (0.3% w/v) at transplanting and one week post transplanting were considered fungicide-protected checks. Pots were placed in a growth room at 24°C temperature and a 16/8 h photoperiod for 12 weeks. Plants were watered on alternate days. Disease developments on each plant were rated every week from 2 weeks after transplanting using the following scale: 0 = no symptoms, 1 = <25% of leaves with symptoms, 2=26 to 50 % of leaves with symptoms, 3 = 51 to 75% of leaves with symptoms, 4 = 76 to 100% of leaves with symptoms and 5= plant dead. The disease index was calculated using the following rating by the formula [22]:

$$\text{Disease index} = \frac{(\text{Rating No.} \times \text{No. of the plants in the rating}) \times 100}{(\text{Total no. of plants} \times \text{highest rating})}$$

Additionally, the number of sclerotia formed on soil surfaces was counted and expressed per cm^2 surface area.

2.11.3 *Field grown plant tests*

The PGPR strains were further evaluated against *S. rolfsii* at the experimental field, Department of Plant Pathology, Bangabandhu Sheikh Mujibur Rahman Agricultural University, Gazipur, Bangladesh. In this experiment, treatments included were an uninoculated negative control, a pathogen inoculated control, a fungicide protected chemical check, and two PGPR treatments. Experiments were laid in a randomized block design (RBD) with three replications for each treatment. The unit plot size was 2.0 m × 2.0 m, and the spacing between plots was 0.5 m. A spacing of 50 cm between rows and plants and a total of 12 plants per plot were assigned. The soil of the field was pulverized well by deep plowing. A recommended dose of 120 kg, 40 kg P,100 kg K, and 2 tons cow dung per hectare was applied. The full dose of P, K, and cow dung and one-third of N was applied at the time of final land preparation, and the remaining N was applied in two equal installments at 3 and 5 weeks after transplanting of tomato. The colonized wheat grain inoculum of *S. rolfsii* was mixed with soil in inoculated micro plots (50 g inoculum/m^2 area). Plots amended with equal amount autoclaved wheat grain were considered as uninoculated negative controls. The soil drenched with Provax 200 (0.3% w/v) at transplanting and one week post transplanting was treated as fungicide-protected check plots. Bacterial treatment of the seedlings was given by dipping roots in the suspension (10^8 CFU mL^{-1}) of *S. maltophilia* PPB3 and *B. subtilis* PPB9 for 30 min before transplanting in the field. The seedlings dipped in the sterilized water for 30 min were considered as unprotected controls. After transplanting, weeding, watering, and other intercultural operations were done regularly. Disease developments on each plant were rated at 12 weeks after transplanting using the following scale: 0 = no symptoms, 1 = <25% of leaves with symptoms, 2=26 to 50 % of leaves with symptoms, 3 = 51 to 75% of leaves with symptoms, 4 = 76 to 100% of leaves with symptoms and 5= plant dead. The disease index was calculated using the following rating by the formula [22]:

$$\text{Disease index} = \frac{(\text{Rating No.} \times \text{No. of the plants in the rating}) \times 100}{(\text{Total no. of plants} \times \text{highest rating})}$$

At the end of the experiment, data on plant height were recorded. Fruits were harvested at several spells and yield was calculated.

2.12 Root Colonization

Studies were conducted to study the root colonization ability of the rhizobacteria in tomato. Seeds were treated and sown in pots as described above. For each treatment, 21 plants were grown in 21 pots for 12 weeks. Roots were harvested at 1, 2, 4, 5, 6, and 7 weeks after sowing. Root systems were thoroughly washed with running tap water to remove adhering soil particles. Roots were cut into top, middle, and bottom regions. Root pieces belong to each segment were weighed and then surface sterilized in 5% (v/v) sodium hypochlorite solution (NaClO) for 1 min followed by several washes in sterile water [23]. Root tissues of each segment were separately homogenized in sterilized distilled water, and appropriate dilutions of the homogenates were plated onto Yeast Peptone Agar (YPA) media. The plates were incubated overnight at 28°C, and the number of colony-forming units (CFU) per gram of root tissue was determined.

2.13 Statistical Analysis

A completely randomized design (CRD) was followed for all experiments. ~~There was a minimum of three replications for each treatment.~~ The data presented are from representative experiments that were repeated at least twice with similar results. Statistix 10.0.0.9 (Analytical Software, FL, USA) was used to compare treatments via ANOVA (analysis of variance) using the least significant differences test (LSD) at a 5% ($P \leq 0.05$) probability level.

3. Results

3.1 *In vitro* antagonistic activities of the bacterial isolates in dual culture assays

In dual culture assays, all the six rhizobacterial isolates screened inhibited the growth of *S. rolfsii*, ranging from 58.22% to 88.29% (Table 1). Of these, maximum inhibition was recorded by isolate *Stenotrophomonas maltophilia* PPB3, followed by isolate *Bacillus subtilis* PPB9 (71.84%). A distinct growth inhibition zone with the pathogen was observed ~~that existed for the test duration~~. In plates treated with Provax 200, a complete inhibition (100%) of the pathogen growth was observed.

3.2 *In vitro* antagonistic activities of the culture filtrates

The cell-free filtrates of all isolates showed effective antagonistic activities against the test pathogen and significantly inhibited the mycelial growth of *S. rolfsii* at all three concentrations compared to control. The inhibition of test pathogen at 10%, 25% and 50% concentrations ranged from 19.75% to

54.43%, 44.24% to 100.00% and 62.89% to 100.00%, respectively. Culture filtrates of strain PPB3 and PPB9 at 25% and 50% concentrations completely (100%) inhibited the mycelial growth of *S. rolfsii*, similar to Provax 200. [illegible]mplete [illegible]ibition ~~of~~ (100%) ~~the mycelial growth~~ of *S. rolfsii* ~~was observed by culture filtrates of strain PPB2 and PPB5 at 50% concentrations~~.

3.3 Inhibition of oxalic acid production by bacteria

The rhizobacteria significantly reduced the oxalic acid production of *S. rolfsii* in dual broth cultures. In the absence of rhizobacteria, *S. rolfsii* isolate SR-1 produced 3.91 mg oxalic acid/ml of culture filtrate, while the quantity of oxalic acid production ranged from 0.77 to 2.14 mg/ml of culture filtrate in dual broth cultures with the antagonists. Of the five strains, the lowest quantity of oxalic acid (0.77 mg/ml) was recorded in dual culture with PPB3 followed by PPB9 (0.83 mg/ml). Since the two isolates, PPB3 and PPB9, and their culture filtrates exhibited the highest inhibition of mycelial growth and maximum degradation of oxalic acid production by the test pathogen, they were selected as elite antagonists against *S. rolfsii* for further studies.

3.4 *In vitro* plant-growth-promoting and biocontrol traits of elite antagonists

The results of Table 2 revealed that multiple plant-growth-promoting and biocontrol activities were displayed by both *S. maltophilia* PPB3 and *B. subtilis* PPB9. Phosphate solubilization was noted with both isolates, while *S. maltophilia* PPB3 exhibited the highest phosphate solubilization. The IAA production by *S. maltophilia* PPB3 and *B. subtilis* PPB9 ranged from 18.47 to 36.24 µg/ml. Both isolates showed growth in an N_2-free medium. Likewise, siderophores were produced by both isolates. Production of HCN and chitinase was detected in PPB3 and PPB9. Both *S. maltophilia* PPB3 and *B. subtilis* PPB9 used ACC as a nitrogen source, implying that these isolates were ACC deaminase positive. Both isolates produced biofilm on glass tube surfaces, although variably. As compared to that of *B. subtilis* PPB9, a higher amount of adherence was observed by the isolate *S. maltophilia* PPB3. Both isolates were antagonistic and inhibited multiple plant pathogenic fungi; *R. solani*, *P. capsici*, and *S. sclerotiorum*.

3.5 *In vitro* effect of rhizobacteria on seed germination and seedling vigor of tomato

The *in vitro* tests showed that the seed treatments with PGPR strains had improved seed germination and seedling vigor than the control [Fig. 1A]. The germination percent of tomato seeds treated with *S.*

maltophilia PPB3 and *B. subtilis* PPB9 was enhanced by 15.11% and 20.27%, respectively, over the value recorded with control. Moreover, the germination appeared to be faster in PGPR-treated seeds than control seeds (data were not shown). The seedling length was significantly higher in seedlings from PPB3 and PPB9-treated seeds, resulting in a 23.99% and 32.16% increase, respectively, compared to control. Seed bacterization with strain PPB3 and PPB9 also significantly improved the vigor index of tomato seedlings. The vigor index of tomato seedlings ranged from 1719 to 1081, where the highest seedling vigor index was recorded in seed bacterization by *B. subtilis* PPB9, and the lowest was in control. These findings indicate that seed biopriming with *S. maltophilia* PPB3 and *B. subtilis* PPB9 is a potentially effective treatment for promoting germination and vigor in tomatoes. Germination and vigor improvement is considered a vital selection trait for biological inoculants, as it offers an early advantage to the plant that could be vital for its subsequent development.

3.6 *In vivo* effect of rhizobacteria on germination and growth of tomato

Improvement of germination was significantly observed in pot experiments with both rhizobacteria treatments in tomato compared to non-inoculated controls. Seeds treated with rhizobacteria resulted in quicker and higher germination than non-treated seeds. Tomato seeds sown in control pots started germination five days after sowing, while 1-2% of PPB3 and PPB9-treated seeds germinated four days after sowing. On average, 63% of tomato seeds germinated in the control treatment 12 days of sowing [Fig. 1B], while both bacterial treatments showed similar germination curves, significantly promoting higher germination compared to the control. Inoculation with strain PPB3 and PPB9 significantly enhanced germination by 25% and 38%, respectively, over the value recorded with control.

Seed bacterization with PPB3 and PPB9 also positively affected plant growth, resulting in significant differences in shoot and shoot growth in the inoculated plants relative to the control. Treatment with PPB3 and PPB9 increased shoot length by 23% and 29%, shoot fresh biomass by 42% and 77%, shoot dry biomass by 61% and 76%, root length by 25% and 48%, fresh root biomass by 53% and 73%, dry root biomass by 43% and 68%, respectively (Table 3). The leaf number was significantly higher in plants inoculated with strains PPB3 (19%) and PPB9 (32%) compared to control. The SLA increased by 35% and 41% due to treatment with PPB3 and PPB9, respectively. Total leaf chlorophyll content

significantly increased by 49% and 53% in plants from seeds treated with PPB3 and PPB9, respectively (Table 3).

3.7 Effect of PGPR on N, P, and K

In general, PGPR increased the concentration of all nutrients except Fe in tomato leaves. At the same time, the significant effect of PGPR treatment in comparison to control was only observed for N, P, and K concentrations. Seed bacterization with PPB3 significantly enhanced N, P, and K concentrations by 48%, 36%, and 29%, respectively, over the control (Table 4). Similarly, the concentrations of N, P, and K were significantly increased by 73%, 64%, and 68%, respectively, in PPB9-treated plants compared to the control plants. Concentrations of Ca2+, Mg2+, Na+, and Fe did not vary significantly between PGPR treatments and control.

3.8 Biocontrol efficacy of PGPR against *Sclerotium rolfsii*

3.8.1 Seedling assays

The results in Figure 2 show that the development of damping-off was fastest in control (unprotected) seedling populations of tomato inoculated with *S. rolfsii*. At the end of the assays, about 74% of the control seedlings had been infected by damping-off [Fig. 2A]. Seed bacterization with *S. maltophilia* PPB3 and *B. subtilis* PPB9 resulted in significant control of the pathogen, causing a delay in symptom development accompanied by a lower number of seedlings with damping-off symptoms. Damping-off symptoms were observed from seeding until the fourth-week post-sowing. At the end of the experiment, there were 77.15% and 64.93% reductions in damping-off infection by *S. maltophilia* PPB3 and *B. subtilis* PPB9, respectively, over unprotected controls. Additionally, a parallel decrease in the number of *S. rolfsii* sclerotia formed on the surfaces of the soils was observed in bacteria-protected seed trays (PPB3; 2.74/cm^2 and PPB9; 4.97/cm^2) compared to unprotected controls (15.26/cm^2) [Fig. 2B]. The disease and sclerotia also developed at the lowest rate in inoculated plants treated with Provax 200. No symptom of damping-off developed in the seedlings grown from Provax 200 treatments until the sixth-week post-sowing. At the seventh week post-sowing, only 4.45% of seedlings from Provax 200 treatments showed damping-off symptoms. The lowest number of sclerotia (0.85/cm^2) was formed on soil surfaces in Provax 200-treated seed trays [Fig. 2B].

3.8.2 Potted assays

Control tomatoes (unprotected) inoculated with *S. rolfsii* showed the first development of southern blight disease, which appeared at the third week (5-week age) post-transplanting [Fig. 3A] and increased progressively with time. Most of the plants were severely affected by *S. rolfsii*, and the disease index was recorded to be 91% at the tenth week (12-week age) post-transplanting. Moreover, a significant and steady increase in the numbers of sclerotia was observed in unprotected inoculated plants, rising from about 1 per cm^2 to 21 per cm^2 soil surface within seven weeks [Fig. 3B]. In contrast, the introduction of PPB3 and PPB9 into the root system effectively controls the disease. The average disease index of PPB3 and PPB9-treated plants with the fungal infection was 29% and 44%, respectively. The percent protection achieved by PPB3 and PPB9 treatment against the disease over control was 67% and 51%, respectively. Moreover, there was a parallel decrease in the number of *S. rolfsii* sclerotia formed on the surfaces of the soils in bacteria-protected plants (PPB9; 2.12/cm^2 and PPB9; 5.60/cm^2 soil surface [Fig. 3B]. The inoculated plants treated with Provax 200 showed no symptoms of disease and suffered no mortality. The fungicide-protected plants remained healthy throughout the experiment. No formation of sclerotia was observed on the surface of the soil drenched with Provax 200.

3.8.3 Field assays

The results of field-grown tomatoes showed that Southern blight disease appeared faster in plants grown in inoculated control plots than in those grown in other plots. The disease symptoms were seen in the field within two weeks of inoculation in inoculated control (unprotected) plants and increased progressively with time. The disease index in these plants was estimated to be about 57.05 at 12-week post-inoculation. On the other hand, inoculated plants were protected significantly with *S. maltophilia* PPB3 and *B. subtilis* PPB9, showing the first symptoms of southern blight within four weeks of inoculation. The protected plants remained almost healthy throughout the experiment and suffered less mortality, recording a disease index of 17.34 and 23.83 in plants treated with *S. maltophilia* PPB3 and *B. subtilis* PPB9, respectively, at 12-week post-inoculation. The percent protection achieved by PPB3 and PPB9 against the disease over inoculated controls was about 69% and 58%, respectively. In addition, these plants were significantly taller (PPB3; 47.58 cm and PPB9; 44.09 cm) and set higher fruit yield (PPB3; 27.87 t/ha and PPB9; 20.81 t/ha) than the pathogen inoculated control (Height- 31.26

cm; Yield- 11.53 t/ha) (Table 5). On the other hand, inoculated plants protected with Provax 200 showed the least disease index (9.78) of southern blight throughout the experiment and suffered no mortality. These fungicide-protected plants were also significantly taller and gave higher fruit yield (Table 5) than the inoculated control plants. This shows that *S. maltophilia* PPB3 and *B. subtilis* PPB9 are potential biocontrol agents that can protect against southern blight disease and improve the yield of tomato plants.

3.9 Tomato Root Colonization by PGPR strains

The root colonization assays showed that both rhizobacteria successfully colonized the roots of tomato plants. The total root population densities of *B. subtilis* PPB9 and *S. maltophilia* PPB3 were 47× 10^7 and 25× 10^7 cfu/g root fresh weight, respectively, during the first week of seedling growth [Fig. 4]. However, the total root population densities of B. *subtilis* PPB9 and *S. maltophilia* PPB3 reached 240× 10^7 and 138× 10^7 cfu/g roots fresh weight, respectively, after seven weeks [Fig. 4]. For both bacteria, root population densities were significantly higher in the bottom part than the middle and upper part. The bacterial population differences among the three root segments increased with the increase of plant age and total bacterial population. At seven weeks, the root population of PPB9 in the bottom root parts was two and four folds higher than in the middle and upper root parts, respectively. Similarly, the bottom root parts had two and three folds higher PPB3 population at the same growth stage than the middle and upper root parts, respectively.

4 Discussion

The exploitation of the rhizobacteria that beneficially influence the growth, development, and protection of plants against diseases facilitates sustainable approaches in agriculture [24-26]. In this study, the two potential strains of rhizobacterium *S. maltophilia* PPB3 and *B. subtilis* PPB9 showed significant antagonistic activity against *S. rolfsii* in several *in vitro* dual culture tests using the bacteria and culture filtrates. Tomato seed bacterization with these bacteria promoted rapid and even seed germination and improved seedling vigor. The introduction of bacteria into the root systems effectively enhanced the plant growth and leaf photosynthetic pigments of tomatoes. The bacteria in this study also appeared to have significant value as efficient biocontrol agents to protect seedlings, potted plants, and field-grown tomatoes against *S. rotfsii*. Both *S. maltophilia* PPB3 and *B. subtilis* PPB9 contributed significantly to

the plant-growth promotion, fruit formation, and yield improvement in tomatoes. Bacteria within the genus *Bacillus* have often been illustrated as effective PGPR, and biological control agents of plant diseases in various crop species, including tomato [12, 27-33], but references to the genus *Stenotrophomonas* is less common in tomato. These results confirm that *Stenotropho[illegible]as* can be a potential PGPR and biological control agent of *S. rotfsii* in tomatoes.

Improvement of germination, seedling vigor, plant growth, and yield by *S. maltophilia* PPB3 and *B. subtilis* PPB9 in this study can be attributed to the enhanced IAA production, ACC-deaminase activity, photosynthesis, and nutrient mobilization. IAA, the major auxin, is a crucial phytohormone produced by many PGPR and seed treatment with such IAA-producing rhizobacteria promotes germination and plant growth [12, 34-36]. The rhizobacterial isolates listed in the present study were capable of producing IAA, ranging from 18.47 to 36.24 µg/ml culture medium. IAA is shown to be involved in the early stages of seed germination [37-38]. It is also found that exogenous IAA treatment increases germination rate, seedling length, and seedling weight in various plant species [39-40]. Seed vigor and germination are directly related to membrane system integrity, particularly mitochondrial repair [41]. IAA is suggested to repair the membrane system integrity and improve the properties of seed vigor and germination [42]. IAA is also known to affect plant growth by promoting root development and apical dominance [43].

In vitro ACC-deaminase activity was shown by the rhizobacterial isolates used in the present study. ACC-deaminase is a crucial enzyme found in some PGPR. Being an immediate biosynthetic precursor of ethylene, ACC directly regulates ethylene production in higher plants [44]. The ACC-deaminase enzyme present in some PGPR breaks down ACC into ammonia and α-ketobutyrate, reducing the substrate for ethylene production and stimulating plant growth by minimizing the ethylene-induced plant growth inhibition [45]. Plant growth-promoting *B. subtilis* and *S. maltophilia* with ACC deaminase activity was previously reported [46-47]. The ACC-deaminase producing PGPR have also shown a significant improvement in the plant photosynthetic apparatus (leaf chlorophyll). The higher photosynthetic potential may lead to higher carbon assimilation and stimulated growth in plants [25].

The tested bacteria showed *in vitro* phosphate solubilization and N_2 fixation ability. The two traits have been known as the potential plant growth-promoting mechanisms by many PGPR [48]. This is supported by the fact that the PGPR inoculation improved concentrations of plant nutrient elements such as N, P, and K in the present study. The increase in N, P, and K concentration in tomato leaves could additionally be explained by one or more indirect PGPR actions such as (1) increase in root length and root surface area for nutrient uptake, (2) activation of different metabolic processes that mobilize nutrients, (3) sorption equilibrium shift and increase in net nutrient transfer into the soil solution, or (4) microbial biomass output in the rhizosphere [49-50].

Inhibition of *S. rolfsii* growth and disease severity by *S. maltophilia* PPB3 and *B. subtilis* PPB9 in this study can be attributed to the production of siderophores, a cell-wall-degrading enzyme such as chitinase, HCN, and decrease of oxalic acid production by *S. rolfsii*. Siderophores producing PGPR are recognized as resilient antagonists against phytopathogenic fungi [51]. Bacterial siderophores deprive plant pathogen of iron under iron-limiting conditions, resulting in plant-growth promotion through pathogen control [52]. Similarly, the volatile antibiotic HCN is accounted for substantial biocontrol of phytopathogenic fungi by antagonists [53-54]. Chitinolytic enzymes such as chitinases that are produced by antagonists break down chitin, a principal constituent of hyphae of pathogenic fungi [55]. Oxalic acid is one of the critical determinants of virulence and pathogenicity of *S. rolfsii* [56]. In this study, oxalic acid production by *S. rolfsii* was significantly inhibited by *S. maltophilia* PPB3 and *B. subtilis* PPB9. Neutralizing this pathogenicity factor of *S. rolfsii* by antagonists may lead to disease management.

Several mechanisms may interpret the disease suppression ability of the tested bacteria. The siderophores produced by bacteria may directly influence the synthesis of different antimicrobial compounds by enhancing the iron-bearing mineral availability to the bacteria [52]. Apart from siderophores, chitinase and HCN produced by antagonists might have a role in disease control. Based on the studies on HCN-producing pseudomonads (HPP), Voisard et al. [54] concluded that HCN was toxic to plant pathogen. Similarly, the chitinase producing PGPR plays a significant role in the biocontrol of phytopathogenic fungi. Antibiotics produced by the rhizobacteria could also act against

the pathogen. These indicate that the biocontrol potential of *B. subtilis and S. maltophilia* against *S. rolfsii* might be due to the activity of one or more antimicrobial compounds produced by these bacteria.

The rhizobacterial antagonists showed early establishment on the tomato roots, contributing to rapid colonization of the rhizosphere and the rhizoplane. There was an increase in the abundance of the antagonists from 25-47× 10^7 to about 138-240× 10^7 colony-forming units per g root tissues within the seven weeks of the experiment. The maximum density of bacteria appeared higher at the root tip. The root tip or root growth zone is particularly active in nutrient uptake and may provide a favorable niche for profuse microbial growth and activity [57-59]. These antagonists also showed consistency in forming a biofilm, which enabled them to make sufficient attachment to the root surface and exert plant growth promotion and biocontrol activities [60]. Earlier studies have also established the role of biofilm and consequential root colonization of PGPR in promoting plant growth and controlling the disease [61-62].

Overall, our results suggest that *S. maltophilia* PPB3 and *B. subtilis* PPB9 are promising bioagents for stimulating growth, managing *S. rolfsii* and, enhancing yields in tomatoes. Introducing these PGPR strains in bio-fertilization and biocontrol could have a positive impact on tomato cultivation.

Acknowledgment

The authors would like to acknowledge the Ministry of Science and Technology, Government of the People's Republic of Bangladesh, to fund this research.

Conflict of interest

The authors declare that they have no conflict of interest.

References

[1] FAOSTAT (2011). http://faostat3.fao.org/faostat-gateway/go/to/download/Q/QC/E

[2] Bhutani RD, Kallo G (1983) Genetics of carotenoids and Iycopen in tomato (*L. esculentum* Mill). *Genetic Agrar* 37: 1- 6

[3] Hwang ES, Bowen PE. (2002) Can the consumption of tomatoes or lycopene reduce cancer risk? *Integr Cancer Ther* 1:121–132.

[4] Blum A, Merei M, Karem A (2006) Effects of tomatoes on the lipid profile. *Clin Invest Med* 29:298- 300.
[5] Silaste ML, Alfthan G, Aro A (2007) Tomato juice decreases LDL cholesterol levels and increases LDL resistance to oxidation. *Br J Nutr* 98:1251- 1258.
[6] Zalom FG (2003) Pests, endangered pesticides and processing tomatoes. *Acta Hortic* 613:223-233.
[7] Mullen J (2001) Southern blight, Southern stem blight, White mold. *The Plant Health Instructor* 2001
[8] Kwon JH, Chi TTP, Park CS (2009) Occurrence of fruit rot of melon caused by *Sclerotium rolfsii* in Korea. *Mycobiol* 37:158-9; http://dx.doi. org/10.4489/MYCO.2009.37.2.158
[9] De Miccolis ARM, Rotolo C, Masiello M, et al. (2014) Occurrence of fungicide resistance in populations of *Botryotinia fuckeliana* (*Botrytis cinerea*) on table grape and strawberry in southern Italy. *Pest Manag Sci* 70:1785–96.
[10] Hossain MM, Sultana F, Islam S (2017) Plant Growth-Promoting Fungi (PGPF): Phytostimulation and Induced Systemic Resistance. In: Singh D, Singh H, Prabha R. (eds) Plant-Microbe Interactions in Agro-Ecological Perspectives. Springer, Singapore
[11] Hossain MM, Sultana F (2020) Application and mechanisms of plant growth promoting fungi (PGPF) for phytostimulation. In: Das SK, Editor. Organic Agriculture, ISBN 978-1-78984-669-0. IntechOpen, London, UK, 10.5772/intechopen.92338
[12] Islam S, Akanda AM, Prova A, et al. (2016) Isolation and identification of plant growth promoting rhizobacteria from cucumber rhizosphere and their effect on plant growth promotion and disease suppression. *Front Microbiol* 6:1360.
[13] Paramasiva M., Mohan S., Muthukrishnan N. & Chandrasekaran A. (2013) Degradation of oxalic acid (OA) producing *Sclerotium rolfsii* (Sacc.) by organic biocides. Archives of Phytopathology and Plant Protection, 46:3, 357-363, DOI: 10.1080/03235408.2012.740983
[14] Hossain MM, Sultana F (2018) Methods for the Characterization of Plant-Growth Promoting Rhizobacteria. In: Medina C, López-Baena F. (eds) Host-Pathogen Interactions. Methods in Molecular Biology, Vol-1734. Humana Press, New York, NY.
[15] Ker K (2011) A greener grass: improving biofuel feedstock production of switchgrass (*Panicum virgatum* L.) by inoculation with endophytic rhizobacteria. St W, Montreal, QC: McGill University.
[16] Homthong M, Kubera A, Srihuttagum M, et al. (2016). Isolation and characterization of chitinase from soil fungi, *Paecilomyces* sp. *Agric Nat Resour*, 50:232–242.
[17] Nautiyal CS, Dasgupta SM, Singh, HB, et al. (2006) Synergistic bioinoculant composition comprising bacterial strains of accession Nos. NRRLB-30486, NRRL B-30487 and NRRL B-30488 and method of producing said composition thereof. US Patent 7097830.
[18] Haque MM, Hirata H, Tsuyumu S (2012) Role of PhoP-PhoQ two-component system in pellicle formation, virulence and survival in harsh environments of *Dickeya dadantii* 3937. *J Gen Plant Pathol* 78:176–189. 10.1007/s10327-012-0372-z
[19] Molazem D, Qurbanov EM, Dunyamaliyev SA (2010) Role of proline, Na and chlorophyll content in salt tolerance of corn (*Zea mays* L.). *American-Eurasian J Agric & Environ Sci* 9(3): 319-324.
[20] Kirk PL (1950) Kjeldahl method for total nitrogen. *Anal. Chem* 22:354–358.
[21] Sundar AR, Das ND, Krishnaveni D (1995) *In vitro* antagonism of *Trichoderma* spp. against two fungal pathogens of Castor. *Indian J Plant Protec* 23(2): 152-155.
[22] Liu L, Kloeppper JW, Tuzun S (1995) Induction of systemic resistance in cucumber against Fusarium wilt by plant growth-promoting rhizobacteria. *Phytopathology* 85:695-8; http://dx.doi.org/10.1094/Phyto-85-695

[23] Hossain MM, Sultana F, Kubota M, Hyakumachi M (2008) Differential inducible defense mechanisms against bacterial speck pathogen in *Arabidopsis thaliana* by plant-growth-promoting-fungus *Penicillium* sp. GP16-2 and its cell free filtrate. *Plant Soil* 304 227–239. 10.1007/s11104-008-9542-3

[24] Hossain MM, Sultana F (2020) Application and mechanisms of plant growth promoting fungi (PGPF) for phytostimulation. In: Das SK, Editor. Organic Agriculture, ISBN 978-1-78984-669-0. IntechOpen, London, UK, 10.5772/intechopen.92338

[25] Hossain MM, Sultana F, Islam S (2017) Plant Growth-Promoting Fungi (PGPF): Phytostimulation and Induced Systemic Resistance. In: D.P. Singh et al. (eds.), Plant-Microbe Interactions in Agro-Ecological. Springer Nature Singapore Pte Ltd.

[26] Vyas P, Rahi P, Chadha BS, et al. (2014) Statistical optimization of medium components for mass production of plant growth-promoting microbial inoculant *Pseudomonas trivialis* BIHB 745 (MTCC5336) *Indian J Microbiol* 54(2):239–241. doi: 10.1007/s12088-013-0425-9.

[27] Rahman MF, Akanda AM, Eivy FZ et al. (2017) Effect of plant growth promoting rhizobacteria on seed germination and growth of wheat plants. *Ann Bangladesh Agric* 21 (1 & 2): 61-71.

[28] Rahman MF, Akanda AM, Eivy FZ et al. (2018) Effect of *Bacillus amyloliquefaciens* on plant growth and suppression of Bipolaris leaf blight in wheat. *Ann Bangladesh Agric* 22 (2): 9-19.

[29] Sultana F, Islam MR, Sabbir MA, et al. (2020) Plant growth promotion and suppression of damping off in tomato by plant growth promoting rhizobacterium *Bacillus amyloliquifaciens*. *Canadian Journal of Agriculture and Crops* 5 (1):59-68

[30] Cendales TC, González CAR, Villota et al. (2017) *Bacillus* effect on the germination and growth of tomato seedlings (*Solanum lycopersicum* L). *Acta Biol Colomb* 22(1):37-44. DOI: http://dx.doi.org/10.15446/abc.v22n1.57375

[31] Fernando WGD, Nakkeeran S, Zhang Y, et al. (2007) Biological control of *Sclerotinia sclerotiorum* (Lib.) de Bary by *Peudomonas* and *Bacillus* species on canola petals. Crop Protection 26: 100-107.

[32] Messiha NAS, van Diepeningen AD, Farag NS, et al. (2007) *Stenotrophomonas maltophilia*: a new potential biocontrol agent of *Ralstonia solanacearum*, causal agent of potato brown rot. *Eur J Plant Pathol* 118:211–225. 10.1007/s10658-007-9136-6

[33] Abeysinghe S (2009) The effect of mode of application of *Bacillus subtilis* CA32r on control of *Sclerotium rolfsii* on *Capsicum annuum*. *Archives of Phytopathology and Plant Protection* 42: 835-846

[34] Prashanth S, Mathivanan N (2010) Growth promotion of groundnut by IAA producing rhizobacteria *Bacillus licheniformis* MML2501. *Archives of Phytopathology and Plant Protection* 43:2, 191-208, DOI: 10.1080/03235400802404734

[35] Spaepen, S., Vanderleyden, J., 2011. Auxin and plantmicrobe interaction. Cold Spring Harb Perspect Biol., 3: a001438 First published online November 17, 2010 doi: 10.1101/cshperspect.a001438

[36] Cordero I, Balaguer L, Rinco´n A, et al. (2018) Inoculation of tomato plants with selected PGPR represents a feasible alternative to chemical fertilization under salt stress. *J Plant Nutr Soil Sci* 181:694-703

[37] Slavov S, van Onckelen H, Batchvarova R, Atanassov A, Prinsen E. 2004. IAA production during germination of Orobanche spp. seeds. J Plant Physiol. 161:847853.

[38] Pieruzzi FP, Dias LLC, Balbuena TS, Santa-Catarina C, dos Santos ALW, Floh EIS. 2011. Polyamines, IAA and ABA during germination in two recalcitrant seeds: Araucaria angustifolia (Gymnosperm) and Ocotea odorifera (Angiosperm). Ann Bot. 108:337345.

[39] Naidu CV, Rajendrudu G, Swamy PM. 2000. Effect of plant growth regulators on seed germination of Sapindus trifoliatus Vahl. Seed Sci Technol. 28:249252.

[40] Tomar A. 2008. Effect of salicylic acid, L-arginine monohydrochloride, gibberellic acid, maleic hydrazide and indole acetic acid on seed germination and seedling growth of Albizzia lebbeck (L.). Plant Arch. 8:495496.
[41] Black M, Bewley JD. 2000. Seed technology and its biological basis. Boca Raton: CRC Press, p. 287316.
[42] Zhao G, & Zhong T (2013) Influence of exogenous IAA and GA on seed germination, vigor and their endogenous levels in *Cunninghamia lanceolata*, Scandinavian Journal of Forest Research, 28:6, 511-517, DOI: 10.1080/02827581.2013.783099
[43] Contreras-Cornejo HA, Macías-Rodríguez LI, Cortés-Penagos C, et al. (2009) *Trichoderma virens*, a plant beneficial fungus, enhances biomass production and promotes lateral root growth through an auxin-dependent mechanism in Arabidopsis. *Plant Physiology* 149:1579-1592
[44] Glick BR (2005). Modulation of plant ethylene levels by the bacterial enzyme ACC deaminase. FEMS Microbiol. Lett. 251, 1–7.
[45] Mayak S, Tirosh T, Glick BR (2004). Plant growth-promoting bacteria confer resistance in tomato plants to salt stress. *Plant Physiol Biochem*, 42, 565–572.
[46] Ghosh, S., Penterman, J.N., Little, R.D., Chavez, R. et al., 2003. Three newly isolated plant growth-promoting Bacilli facilitate the seedling growth of canola, Brassica campestris. Plant Physiol. Biochem., 41, 277–281.
[47] Singh RP, Jha PN. (2017) The PGPR Stenotrophomonas maltophilia SBP-9 Augments Resistance against Biotic and Abiotic Stress in Wheat Plants. Front Microbiol. 2017;8:1945. Published 2017 Oct 9. doi:10.3389/fmicb.2017.01945
[48] Islam MT, Hossain MM (2012) Plant probiotics in phosphorus nutrition in crops, with special reference to rice. In: Maheshwari DK (ed.) Bacteria in Agrobiology: Plant Probiotics. Springer-Verlag Berlin Heidelberg. pp. 325-363.
[49] Richardson AE, Barea JM, McNeill AM, et al. (2009) Acquisition of phosphorus and nitrogen in the rhizosphere and plant growth promotion by microorganisms. *Plant Soil* 321: 305– 339.
[50] Spaepen S, Vanderleyden J, Okon Y (2009) Plant growth- promoting actions of rhizobacteria. *Adv Bot Res* 51:283– 320.
[51] Chaiharn M, Chunhaleuchanon S, Lumyong S (2009) Screening siderophore producing bacteria as potential biological control agent for fungal rice pathogens in Thailand. World J Microbiol Biotechnol 25:1919–1928
[52] Joseph B, Patra RR, Lawrence R (2007) Characterization of plant growth promoting rhizobacteria associated with chickpea (*Cicer arietinum* L.) *Int J Plant Prod* 1:141–152.
[53] Ahmad F, Ahmad I, Khan MS (2008) Screening of free-living rhizospheric bacteria for their multiple plant growth promoting activities. Microbiol Res 163:173–181
[54] Voisard C, Keel C, Haas D, et al. (1989) Cyanide production by *Pseudomonas fluorescens* helps suppress black root rot of tobacco under gnotobiotic conditions. *EMBO J* 8:351–358.
[55] Podile AR, Prakash AP (1996) Lysis and biological control of *Aspergillus niger* by *Bacillus subtilis* AF 1. *Can J Microbiol* 42:533–538. doi: 10.1139/m96-072.
[56] Cessna SG, Sears VE, Dickman MB, Low PS (2000) Oxalic acid, a pathogenicity factor for *Sclerotinia sclerotiorum*, suppresses the oxidative burst of the host plant. Plant Cell 12:2191–2200
[57] Silk WK, Bogeat-Triboulot MB (2014) Deposition rates in growing tis-sue: Implications for physiology, molecular biology, and response to environmental variation. *Plant Soil* 374:1–17. doi:10.1007/s11104-013-1726-9
[58] Hossain MM, Sultana F, Miyazawa M et al. (2014) The plant growth-promoting fungus *Penicillium* spp. GP15-1 enhances growth and confers protection against damping-off and anthracnose in the cucumber. *Journal of Oleo Science* 63(4): 391-400.

[59] Dupuy LX, Silk WK (2016) Mechanisms of early microbial establishment on growing root surfaces. *Vadose Zone J* 15:1–13. doi: 10.2136/vzj2015.06.0094

[60] Rudrappa T, Quinn WJ, Stanley-Wall NR, et al. (2007) A degradation product of the salicylic acid pathway triggers oxidative stress resulting in down-regulation of *Bacillus subtilis* biofilm formation on Arabidopsis thaliana roots. *Planta* 226:283–297. doi: 10.1007/s00425-007-0480-8.

[61] Combes-Meynet E, Pothier JF, Moenne-Loccoz Y, et al. (2011) The Pseudomonas secondary metabolite 2,4-diacetylphloroglucinol is a signal inducing rhizoplane expression of *Azospirillum* genes involved in plant-growth promotion. *Mol Plant Microbe Interact* 24:271–284. doi: 10.1094/MPMI-07-10-0148.

[62] Beauregard PB, Chai Y, Vlamakis H, et al. (2013) *Bacillus subtilis* biofilm induction by plant polysaccharides. *Proc Natl Acad Sci* USA. 2013;110:E1621–E1630. doi: 10.1073/pnas.1218984110.

Figure Legends

Figure 1: Germination percentage and vigor index of tomato seeds treated with PGPR. (A) *In vitro* effect of bacterial seed treatment on germination and Vigor Index of tomato. Bacterized and non-treated tomato seeds were placed on moistened filter paper in 9-cm Petri dishes. Seven days after germination, the germination percentage and seedling length were recorded. Vigor index was calculated from the following formula: Vigor index = % germination × total plant length. (B) *In vivo* effect of bacterial seed treatment on germination of tomato. Bacterized and non-treated tomato seeds were sown in pots, and germination was evaluated daily for 12 days. Values are means ± standard errors. Fisher LSD test (P <0.05) was done to indicate significant differences among treatments.

Figure 2 Development of damping-off and sclerotia in protected and unprotected seedlings of tomato in seed trays. (A) Percentage of seedlings with damping-off symptoms in bacteria-protected (PPB3 and PPB9), unprotected and fungicide-protected seedlings of tomato in seed trays. (B) Abundance of sclerotia of *S. rolfsii* in the soils of bacteria-protected (PPB3 and PPB9), unprotected and fungicide-protected seed trays. Each point in the line represents the mean value within each treatment category. Vertical lines denote the standard errors.

Figure 3 Development of southern blight disease and sclerotia in protected and unprotected tomatoes in pots. (A) Disease index for southern blight disease of infected plants within each treatment category; inoculated control (*S. rolfsii*); inoculated plus *S. maltophilia* PPB3 (*S. roflsii*+ *S. maltophilia* PPB3), inoculated plus B. sublis PPB9 (*S. roflsii*+*B. subtilis* PPB9) and inoculated plus fungicide (*S. roflsii*+Provax 200). Tomato seedlings of each treatment category were grown in pots inoculated with *S. rolfsii* for ten weeks. Disease developments on each plant was rated weekly as described in Materials and Methods. Each point in the line is the mean disease index in 15 plants within each treatment category. Vertical lines denote the standard errors. (B) An abundance of sclerotia of *S. rolfsii*

in the soils of protected and unprotected adult potted plants. Each point in the line is the mean number of sclerotia in 15 pots within each treatment category; inoculated control (*S. rolfsii*); inoculated plus *S. maltophilia* PPB3 (*S. roflsii*+ *S. maltophilia* PPB3), inoculated plus B. sublis PPB9 (*S. roflsii*+*B. subtilis* PPB9) and inoculated plus fungicide (*S. roflsii*+Provax 200). Vertical lines denote the standard errors.

Figure 4 Population of *S. maltophilia* PPB3 and *B. subtilis* PPB9 in the upper (toward stem) (top), middle, and lower (toward root tip) (bottom) segments of 1-, 2-, 3-, 4-, 5-, 6- and 7-week-old tomato seedlings. Data are presented as numbers of CFU g^{-1} fresh weight, each from three sets of 5 roots harvested at 1-, 2-, 3-, 4-, -5, -6 and 7-week after growth.

Table 4. Inhibition of mycelial growth and oxalic acid production of *Sclerotium rolfsii* by bacterial antagonists

Treatments	Inhibition of *S. rolfsii* in dual culture (%)	Inhibition of S. rolfsii1 at different concentrations of culture filtrate of antagonists (%)			Oxalic acid production by *S. rolfsii* (mg/ml)
		10%	25%	50%	
Control	0.00±0.00a	0.00±0.00a	0.00±0.00a	0.00±0.00a	3.91±0.03g
Pseudomonas stutzeri PPB1	58.22±0.96b	19.75±0.13b	44.24±0.14b	62.89±0.65b	2.14±0.01f
Bacillus subtilis PPB2	61.84±0.83c	45.78±0.35e	67.38±0.00d	100.00±0.00d	0.94±0.01c
Stenotrophomonas maltophilia PPB3	88.29 ± 0.51f	53.48±2.18f	100.00±0.00f	100.00±0.00d	0.77±0.01a
B. amyloliquefaciens PPB4	62.82±0.77c	34.29±0.51c	54.34±0.45c	68.57±0.71c	1.98±0.02e
B. subtilis PPB5	66.97±1.48d	37.58±0.53d	70.76±1.01e	100.00±0.00d	1.87±0.01d

B. subtilis PPB9	71.84±0.95e	54.43±0.60f	100.00±0.00f	100.00±0.00d	0.83±0.01b
Provax 200	100.00±0.00g	100.00±0.00g	100.00±0.00f	100.00±0.00d	NT**

*Different letters denote significant differences among treatments for each column according to Fisher's LSD test (*P < 5%*).
**NT= Not tested

Table 2 Plant growth-promoting (PGP) and biocontrol traits of elite bacterial isolates

PGP trait	Bacterial Isolates	
	Stenotrophomonas maltophilia PPB3	*Bacillus subtilis* PPB9
Phosphate solubilization	+++	++
Production of indole 3-acetic acid (μg/ml)	18.47±0.27	36.24±0.63
Nitrogen fixation	++	++
Siderophore	++	++
Chitinase	++	+
Hydrogen cyanide (HCN)	+++	++
1-aminocyclopropane-1-carboxylate (ACC) deaminase	++	+++
Biofilm (OD_{600})	0.18±0.01	0.26±0.02
Antagonism in dual culture against		
Rhizoctonia solani	+++	+++
Phtophthora capsici	+++	++
Sclerotinia sclerotiorum	+++	++

+, positive; −, negative result for the test. For phosphate solubilization, siderophore production and chitinase production: +, zone of clearance <0.2 mm; ++, zone of clearance 0.2–0.4 mm; +++, >0.4 mm. For N_2 fixation: +, growth of bacterium in the N_2-free medium; −, no growth of bacterium in the N_2-free medium. For ACC deaminase production: +++ good; ++ medium; + slight. For antifungal assay: +, zone of inhibition <0.2 mm; ++, zone of inhibition between 0.2 and 0.4 mm; +++, zone of inhibition >0.4 mm

Table 3 Effect of PGPR treatment on the growth of tomato plants in pots

Treatment	Shoot			Root			Leaf		
	Length (cm)	Fresh weight (g)	Dry weight (g)	Length (cm)	Fresh weight (g)	Dry weight (g)	Number	Specific Lear Area (m^2 kg^{-1})	Total Chlorophyll (mg g^{-1} FW)
Control	25.34±0.65a*	8.76±0.51a	0.81±0.02a	15.34±0.47a	1.26±0.03a	0.16±0.01a	4.85±0.12a	22.95±1.26a	20.23±0.53a
S. maltophilia PPB3	31.31±1.35b (23.56%)**	12.45±0.61b (42.12%)	1.31±0.02b (61.73)	19.20±0.56b (25.16)	1.93±0.02b (53.17)	0.23±0.01b (43.75)	5.81±0.07b (21.03)	32.16±2.07b (35.77)	30.18±1.10b (49.18)
B. subtilis PPB9	32.77±0.90b (29.32%)	15.56±0.58c (77.63%)	1.43±0.04c (76.54)	22.71±0.61c (48.04)	2.19±0.04c (73.81)	0.27±0.01c (68.75)	6.42±0.08c (32.37)	32.45±2.68b (41.39)	31.03±0.96b (53.39)

*Different letters denote significant differences among treatments for each row according to Fisher's LSD test (*P < 5%*).
** Data in parenthesis indicate the increase over control.

Table 4 Effects of *S. maltophilia* PPB3 and *B. subtilis* PPB9 strains on nutrient concentrations in tomato leaves

Nutrient	Control	*S. maltophilia* PPB3	*B. subtilis* PPB9
%N	1.68±0.11c*	2.48±0.13b<br>(47.62)**	2.91±0.16a<br>(73.21)
P	2.22±0.18c	3.01±0.26ab<br>(35.59)	3.64±0.28a<br>(63.96)
K	22.16±1.98c	28.65±2.21b<br>(29.29)	37.31±2.24a<br>(68.37)
Ca2+	10.56±1.10ab	11.24±1.16ab<br>(11.73)	11.45±1.19a<br>(13.82)
Mg2+	5.08±0.31ab	5.12±0.48ab<br>(0.79)	5.91±0.56a<br>(16.34)
Na	0.65±0.05ab	0.68±0.05ab<br>(4.62)	0.72±0.06a<br>(10.77)
Fe	0.06±0.01a	0.07±0.01a<br>(16.67)	0.06±0.01a<br>(0.00)

*Data, expressed in mg g^{-1} of dry weight or % for N, are mean values ± standard errors, n = 6–8. Different letters denote significant differences among treatments for each row according to Fisher's LSD test ($P < 5\%$).

** Data in parenthesis indicate the increase over control.

Table 5 Effect of bacterial antagonists on disease severity and yield-contributing parameters in *Sclerotium rolfsii*-inoculated tomato cv. Minto Super F1 in the field

Treatment category	Disease index	Plant height (cm)	Yield (t/ha)
Uninoculated negative control	12.34±0.41b*	41.08±2.14b	21.64±1.37b
Pathogen Inoculated Control (*S. rolfsii*)	57.05±3.17e	31.26±1.19a	11.53±0.63a
S. maltophilia PPB3+ *S. rolfsii*	17.34±1.51c (-69.61%)**	47.58±3.08c (52.21%)	27.87±1.45c (141.72)
B. subtilis PPB9+ *S. rolfsii*	23.83±1.11d (-58.23%)	44.09±2.46b (41.04%)	20.81±1.15b (80.49)
Provax 200+ *S. rolfsii*	9.78±0.33a (-82.86%)	42.89±2.63b (37.20%)	22.92±1.89b (107.46)

*Different letters denote significant differences among treatments for each row according to Fisher's LSD test (*P < 5%*).
** Data in parenthesis indicate the increase or decrease over pathogen inoculated control.

Figure 1

Click here to access/download;Figure;Fig. 1.tif

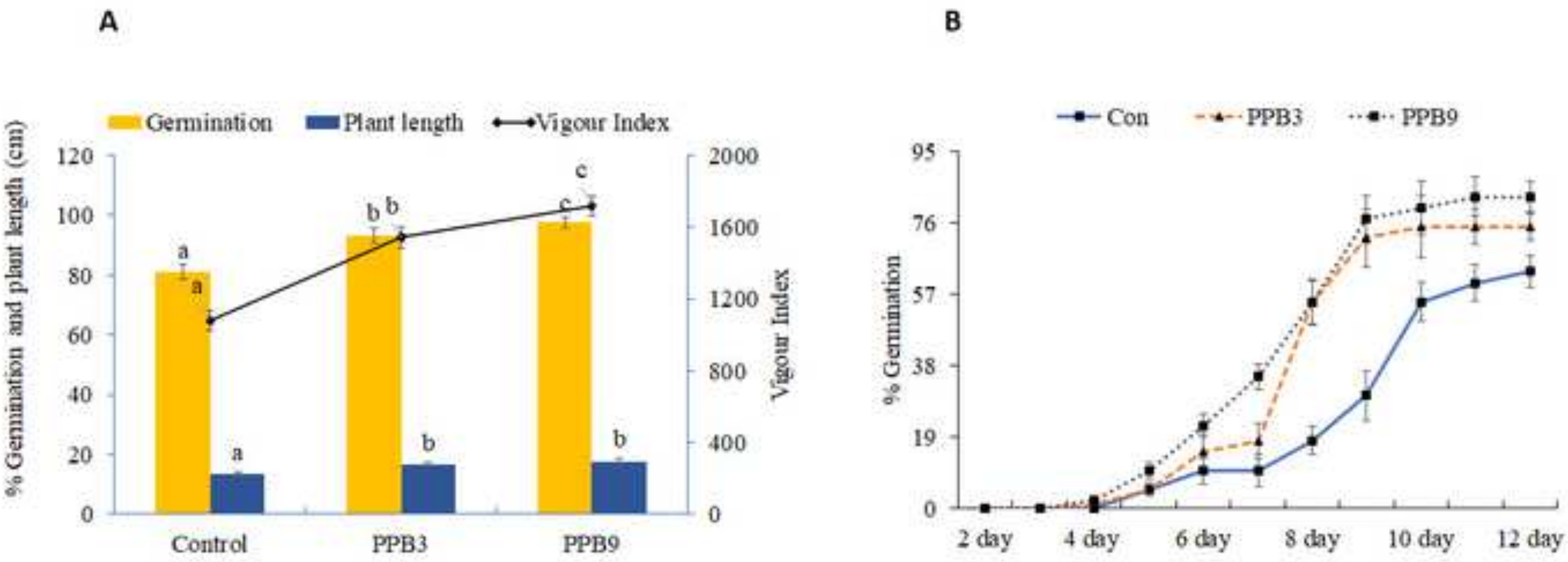

Figure 2

Click here to access/download;Figure;Fig 2R.tif

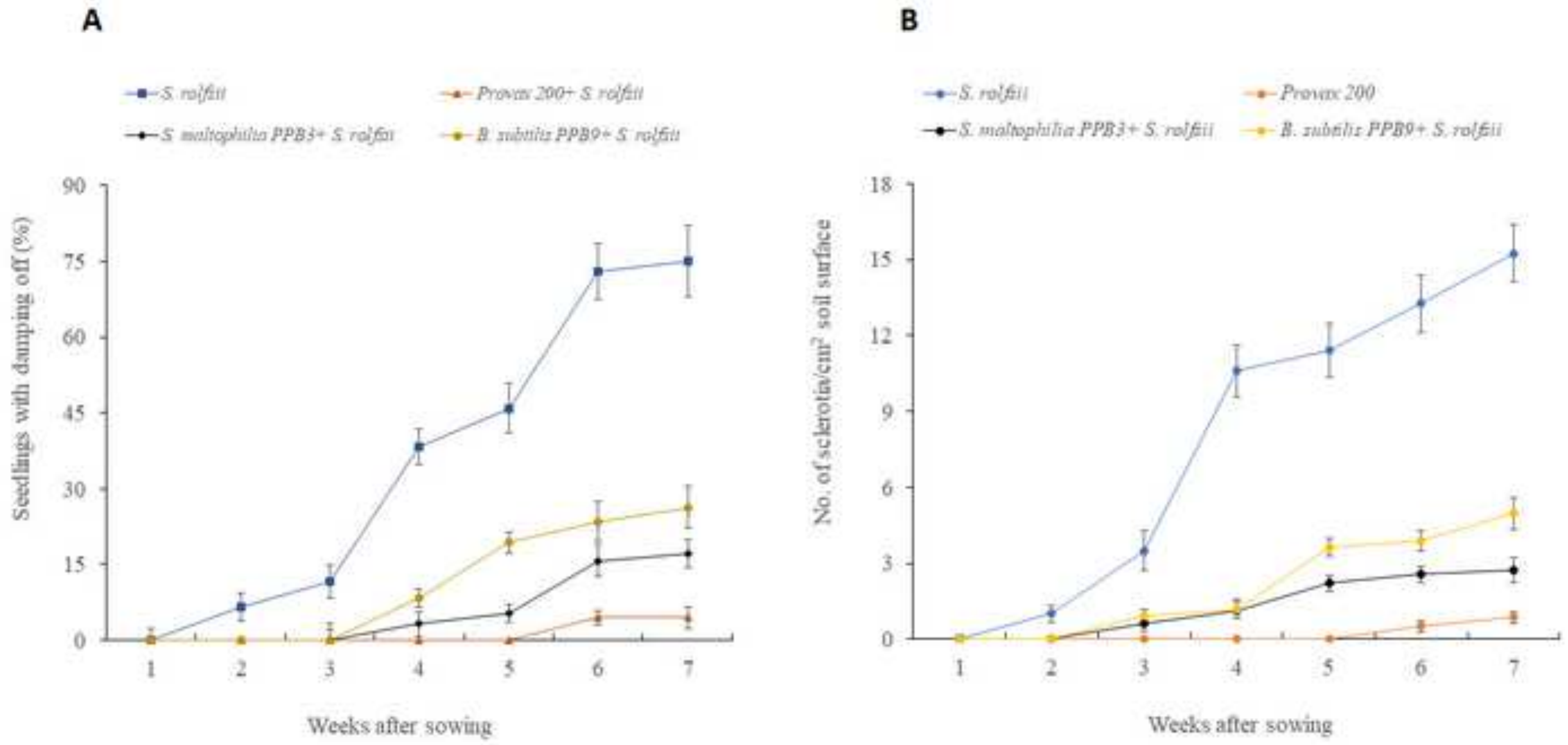

Figure 3

Click here to access/download;Figure;Fig 3R1.tif

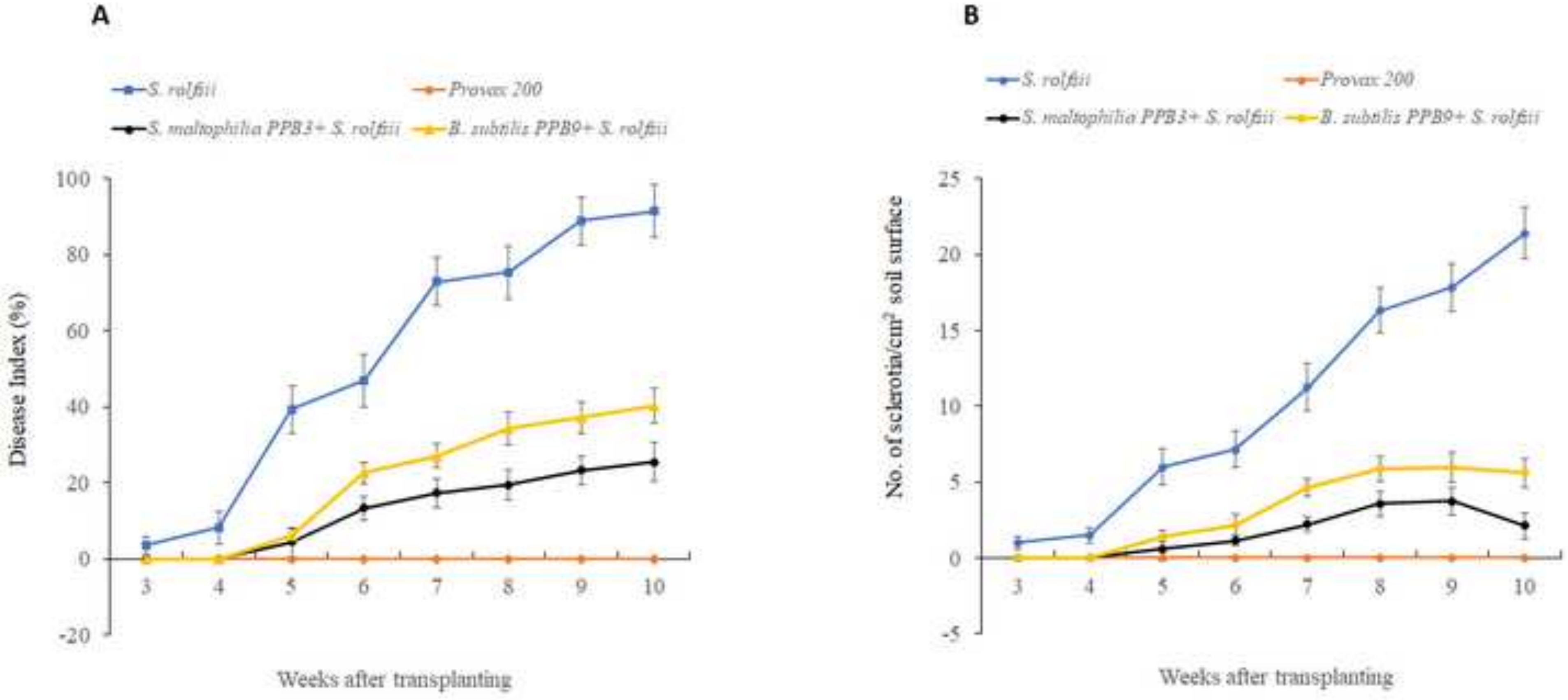

Figure 4

Click here to access/download;Figure;Fig 4.tif

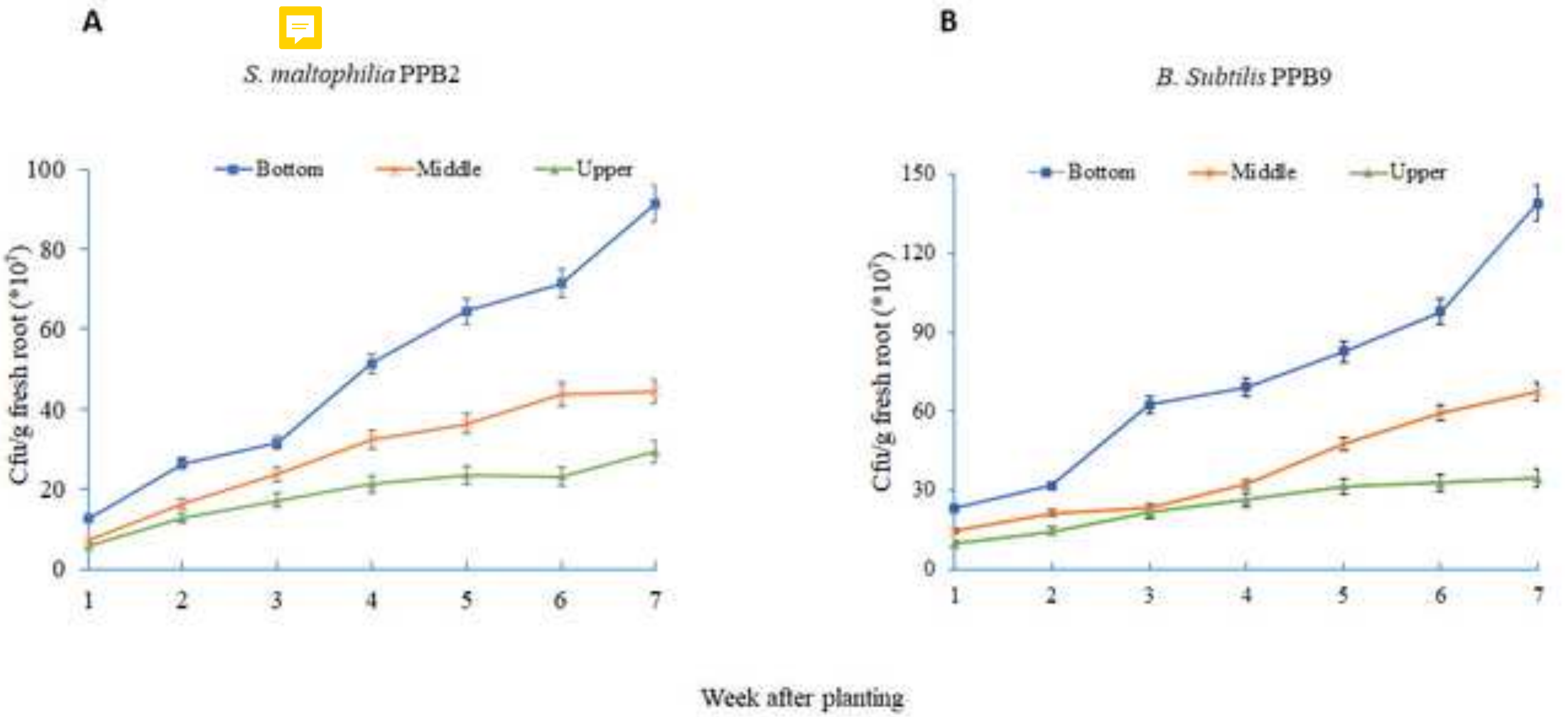

Supplement: Attachment — Submitted filename: PONE-D-21-13656_reviewer.pdf [file pone.0267253.s002.pdf]
