# Supplementary material for: Assessing the potentials of bacterial antagonists for plant growth promotion, nutrient acquisition, and biological control of Southern blight disease in tomato
Source: PLoS One. 2022 Jun 8;17(6):e0267253. doi: 10.1371/journal.pone.0267253 (PMC9176874; doi:10.1371/journal.pone.0267253)
Supplement: Attachment — Submitted filename: Response to Reviewers.docx [file pone.0267253.s003.docx]

**Response to Reviewers**

First of all, we would like to acknowledge the excellent efforts given by the Editor and Reviewers to analyse and evaluate our manuscript. We carefully checked the comments made by Editor and two Reviewers one by one and incorporated the proposed amendments in our manuscript accordingly. The text of any added subject matter is shown by Track changes Mode in Microsoft word.

**Point-by-point responses to the Editor and Reviewer comments.**

**Editor’s comments:**

1.**Please ensure that your manuscript meets PLOS ONE's style requirements, including those for file naming. The PLOS ONE style templates can be found at**

We have followed PLOS ONE’s style and formatted the manuscript accordingly.

**2. Please include a copy of Table 1 which you refer to in your text on page 15. (Please note that two tables in the manuscript have the title of 'table 4'.)**

We have corrected the Table names.

**Reviewer #1:**

We would like to thank you for your kind suggestion. We addressed all your recommendations in the revised manuscript. We believe that your suggestion and comments have improved the manuscript significantly. Please see our response to your specific comment regarding this matter.

1. **Gram-positive bacteria do not grow in classic CAS plates, you tested several Bacillus strains, how did you manage to perform this experiment?**

We have described the experiment elaborately and appropriately in Materials and Methods (Page 10, Line 1-13).

2. **In this section you aim to determine biofilm formation by bacterial isolates. However, the methodology you mentioned is used for pellicle (one type of biofilm) determination, why did you choose this? I think it would be interesting to measure general biofilm formation using crystal violet as described by the same reference authors cited (Haque et al. 2012).**

The bacteria of this study produced only air-liquid biofilm (only form pellicle), not solid-air-liquid biofilm. That is why we did not use the Crystal violet method.

3. **Please specify the concentration (CFU/OD) of the bacterial preparation that you used to inoculate seeds.**

We specified the concentration of the bacterial treatment in the materials and methods (Page 12, Line 16).

4. **Were plants watered using water, or did you use the nutrient solution?**

We used only water regularly but use nutrient solution weekly until eight weeks after planting.

5. **The decrease in the production of oxalic acid with the co-cultures of rhizobacteria and S. rolfsii could be due to growth inhibition of the pathogen or to the consumption of oxalic acid by rhizobacteria. I think it would be interesting to investigate this. For example, you could express the oxalic acid concentration in relation to fungal biomass.**

We agree with you. That is why according to your suggestion, we have expressed the oxalic acid concentration in relation to fungal biomass (Page 8 Line 1-14).

6. **Moreover, you could check if rhizobacteria are able to growth using oxalic acid as unique carbon source.**

We have tested the ability of the two selected rhizobacteria to utilize oxalic acid as a carbon source and added the results in the manuscript. Both rhizobacteria were found to utilize oxalic acid for their growth. (Fig. 1) (page 20 line 12-23)

7. **I would remove the first sentence in this section because it is confused since most of the elements did not change due to inoculation (Fe, Ca, Mg and Na) as authors mentioned in the last sentence of this section.**

We have removed the first sentence (page 26).

8. **Page 11, line 23: remove at 28°C**

We have removed the sentence as it was repeated.

9. **Page 18, line 16: Please include the inhibition % of PPB3**

We have included the % inhibition of PPB3.

10. **Page 12, line 13: vortexing**

We have corrected it.

**Reviewer #2:**

We express sincere gratitude to you for your valuable suggestion. We addressed all your concerns and comments in the revised manuscript. We believe that the quality of the manuscript has been improved substantially by incorporating your suggested revision. Please see our response to your specific comment regarding this matter.

1. **The author did not summarize the main research and key findings well. Example,** **they did not state that five strains were screened from which two strains were selected for superior performance.**

We have summarized the key research findings and included them in the abstract, discussion, and conclusions according to your suggestion.

2. **Why test the filtrates? What is the significance of 20%, 50% etc. filtrate concentration? the discussion did not address the significance of the findings.**

We have discussed the significance of the findings concerning the antagonistic activity of culture filtrates of bacteria according to your suggestion (Page 33, Line 16-22).

3. **Analysis of the effects of S. maltophilia PPB3 and B. subtilis PPB9 strains on nutrient concentrations in tomatoes included N, P, K, Ca2+, Mg2+, Na and Fe, but significant differences were only in NPK. The authors identified other literature but did not explain how this study related to previously published research or the justification of all the studies presented**.

We have improved the discussion by citing related articles according to your suggestion (Page 33, Line 1-15).

4. **Figure and table captions do not stand alone (independent) but are linked to materials and methods, names are not spelt out on Table or figure captions. example B. subtilis.**

We have improved the legends of all figures and Tables.

5. **Qualities of figures need to be improved, legends of figures and tables are not accurate example comparisons within rows instead of columns.**

We have also improved the quality of the figures and amended Table 4 according to your suggestion.

6. **Although the research is technically sound, the presentation need improvement and editing is needed, there are spelling mistakes. It would be difficult for another researcher to reproduce the study with the same methods because the materials do not include enough details, example, what was the environment of the experiment on potted plants? Type of soil, pot size, irrigation, fertilization, temperature is important for this disease. Overall, the writing quality need improvement**

We have thoroughly revised the manuscript and improved the writing quality of the whole manuscript, including Materials and Methods. In addition, we included necessary information about soil type, pot size, irrigation, fertilization and growth conditions.
