# Supplementary material for: Assessing the potentials of bacterial antagonists for plant growth promotion, nutrient acquisition, and biological control of Southern blight disease in tomato
Source: PLoS One. 2022 Jun 8;17(6):e0267253. doi: 10.1371/journal.pone.0267253 (PMC9176874; doi:10.1371/journal.pone.0267253)
Supplement: Attachment — Submitted filename: Response to ReviewersR1.docx [file pone.0267253.s004.docx]

**Response to Reviewers**

First of all, we would like to admit the outstanding efforts given by the Academic Editor and Reviewers to evaluate our manuscript. We have carefully checked the Reviewers comments and made the necessary amendments.

**Point-by-point responses to the Editor and Reviewer comments**

**Reviewers' comments:**

**1. While revising your submission, please upload your figure files to the Preflight Analysis and Conversion Engine (PACE) digital diagnostic tool, https://pacev2.apexcovantage.com/. PACE helps ensure that figures meet PLOS requirements. To use PACE, you must first register as a user. Registration is free. Then, login and navigate to the UPLOAD tab, where you will find detailed instructions on how to use the tool. Please note that Supporting Information files do not need this step.**

We have uploaded our figures in PACE, and PACE has confirmed that the figure file meets PLOS requirements. The PACE processed figure files have been included in the resubmission of the manuscript.
