# Supplementary material for: Assessing the potentials of bacterial antagonists for plant growth promotion, nutrient acquisition, and biological control of Southern blight disease in tomato
Source: PLoS One. 2022 Jun 8;17(6):e0267253. doi: 10.1371/journal.pone.0267253 (PMC9176874; doi:10.1371/journal.pone.0267253)
Supplement: Attachment — Submitted filename: Response to Reviewers.docx [file pone.0267253.s005.docx]

**Responses to Editor’s and Reviewer’s comments to authors**

First of all, we would like to acknowledge the excellent efforts given by the Editor and Reviewers to analyse and evaluate our manuscript. We carefully checked the comments and incorporated the proposed amendments in our manuscript accordingly. The text of any added subject matter is shown by Track changes Mode in Microsoft word.

**Point-by-point responses to the Reviewer comments.**

**Reviewer #1:**

We would like to thank you for your kind suggestion. We have addressed all your recommendations in the revised manuscript. Please see our response to your specific comment regarding this matter.

1. **Page 20, line 5: Please correct the amount of oxalic acid produced in co-cultivate with PBB9, it doesn´t match with the results in Table 1.**

We have corrected it

2. **Figure 2A: You are trying to show 3 different traits (germination, plant length and vigour index) however you have only 2 vertical axes, so you can´t show the reference for plant length so it is a bit confusing. Since you already show germination in the figure 2B, I would remove this parameter from figure 2A, so it is easier to understand.?**

We have amended Figure 2A according to your suggestion.

**Reviewer #2:**

We express sincere gratitude to you for your valuable suggestion. We have addressed your comments in the revised manuscript. Please see our response to your specific comment regarding this matter.

1. **The MS is now modified as suggested but I suggest to reduce number of references.**

Following your suggestion, we have reduced the number of references from 79 to 63.
